# Impacts of soil erosion and climate change on the built heritage of the Pambamarca Fortress Complex in northern Ecuador

**DOI:** 10.1371/journal.pone.0281869

**Published:** 2023-02-23

**Authors:** Fabián Santos, Nora Calle, Santiago Bonilla, Fausto Sarmiento, Mathew Herrnegger

**Affiliations:** 1 Centro de Investigación para el Territorio y el Hábitat Sostenible (CITEHS), Universidad Tecnológica Indoamérica, Machala y Sabanilla, Quito, Ecuador; 2 Departamento de Ciencias de la Computación, Universidad de las Fuerzas Armadas (ESPE), Sangolquí, Ecuador; 3 Geography Department, Neotropical Montology Collaboratory, University of Georgia, Athens, Georgia, United States of America; 4 Department of Water, Atmosphere and Environment, Institute of Hydrology and Water Management, University of Natural Resources and Life Sciences, Vienna, Austria; Arab Academy for Science Technology and Maritime Transport, EGYPT

## Abstract

The Pambamarca fortress complex in northern Ecuador is a cultural and built heritage with 18 prehispanic fortresses known as *Pucaras*. They are mostly located on the ridge of the Pambamarca volcano, which is severely affected by erosion. In this research, we implemented a multiscale methodology to identify sheet, rill and gully erosion in the context of climate change for the prehistoric sites. In a first phase, we coupled the Revised Universal Soil Loss Equation (RUSLE) and four CMIP6 climate models to evaluate and prioritize which *Pucaras* are prone to sheet and rill erosion, after comparing historical and future climate scenarios. Then, we conducted field visits to collect geophotos and soil samples for validation purposes, as well as drone flight campaigns to derive high resolution digital elevation models and identify gully erosion with the stream power index. Our erosion maps achieved an overall accuracy of 0.75 when compared with geophotos and correlated positively with soil samples sand fraction. The *Pucaras* evaluated with the historical climate scenario obtained erosion rates ranging between 0 and 20 ton*ha^-1^*yr^-1^. These rates also varied from -15.7% to 39.1% for four future climate change models that reported extreme conditions. In addition, after identifying and overflying six Pucaras that showed the highest erosion rates in the future climate models, we mapped their gully-prone areas that represented between 0.9% and 3.2% of their analyzed areas. The proposed methodology allowed us to observe how the design of the *Pucaras* and their concentric terraces have managed to reduce gully erosion, but also to notice the pressures they suffer due to their susceptibility to erosion, anthropic pressures and climate change. To address this, we suggest management strategies to guide the protection of this cultural and built heritage landscapes.

## 1. Introduction

Cultural heritage consists of the heritage of tangible and intangible heritage assets of a group or society that is inherited from past generations [1]. They have a diversity of values including symbolic, historic, artistic, aesthetic, ethnological or anthropological, scientific and social significance [2]. Cultural heritage and especially built heritage, which consists of historical layers made of brick, plaster, wood, metal and stone, is frequently exposed to different threats and damages. Their preservation as monuments or a group of buildings and sites is also threatened by the lack of knowledge and management. In Latin America, where 13.2% of World heritage sites are located, Pavlova et al. [3] indicated that this region is severely affected by geohazards. Heritage preservation work and engagement in the region is also obstructed by a deprived education system and low economic status, contributing to deficits in preservation [4]. These challenges are further amplified as on-going climate change will accelerate the decay processes, due to alteration of temperature and precipitation regimes, in addition to more frequent extreme events and changes in climate variability [5]. Therefore, identifying these risks and proposing strategies for climate change adaptation are of prime importance, considering vulnerabilities and challenges for preserving built heritage. This also depends on new methodological approaches, as important historic complexes in Latin America are located along the Andean ridge, where scarce data complicate the prediction of land degradation processes, such as erosion by water [6]. *Pucaras* (or prehispanic fortresses) located in northern Ecuador are important prehispanic and historic infrastructures [7] and are located along the Andean ridge where high erosion rates occur [8]. Identifying erosion hotspots in this area is therefore essential for managing and conserving these historical sites and cultural heritage, which could be at risk due to climate change impacts.

Erosion by water is the surface process that occurs principally in exposed soils, where contributing drainage area, rainfall and surface slope are sufficient to cause transportation and soil incision [9]. Differentiated by relevant processes and magnitude, erosion by water can be categorized into three general types: sheet, rill and gully erosion. Rill and gully erosion are characterized by channel formation of sizes above 150 cm^2^, while sheet erosion only involves losses of topsoil. To predict the first two, different models have been developed, where Revised Universal Soil Loss Equation [RUSLE; 10–14] is probably the most applied worldwide [15,16]. This model relies on information representing the major factors affecting erosion by water, i.e. rainfall, soil texture, topography and land cover and management, to quantify mean annual erosion rates. For its flexibility, the literature recognizes it as a tool for predicting soil erosion due climate change [17], since rainfall data can be derived from global or regional climate models to predict impacts.

Climate models provide several scenarios of possible developments of atmospheric parameters due to different carbon emission pathways. Within the Coupled Model Intercomparison Project (CMIP), different models from numerous research groups can be compared and analyzed together [18]. This is an advance as the evaluation of different models is more suitable for identifying the “worst” and “best” climate change scenario, rather than relying on a single deterministic model run, since large uncertainties exist [19]. Coupling these climate models with erosion models is an opportunity for identifying erosion-prone areas at built heritage sites. Global Circulation models (GCMs) provide climate projections at coarse spatial resolutions (> 100 km) and can be useful for exploring regional patterns of changing climate conditions. At a more detailed spatial resolution, GCMs are uncertain and different procedures for evaluating rain erosion at the field scale are required in the context of built heritage management. Therefore, novel approaches using Unmanned Aerial Vehicles (UAVs or drones) are an interesting opportunity to complement regional assessments and propose on-the-site actions to preserve built heritage exposed to climatic impacts.

In recent years, UAVs have become a popular and economical viable instrument to develop innovative for various applications, including those related to archaeology and heritage site management [20]. Their portability and capability to produce georeferenced orthophotographs and Digital Terrain Models (DTMs) makes them ideal for conducting assessments of built heritage sites in remote areas. In this regard, several studies have documented UAVs applications including seismic capacity assessment of historical buildings [21], rockfall simulation and heritage affectation [22] and even the threat of soil moisture to historical monuments [23]. While many other applications exist, studies focusing on threats related to rain erosion risks [24] are less abundant. These risks can be sufficiently modelled with UAVs, as high spatial resolution DTMs can be used to quantify gullies and badland erosion [25]. This is useful for recommending measures to reduce erosion vulnerability and complement regional assessments focusing on rain erosion and climate change.

For these reasons, the main objective of this research was to development a multiscale methodology coupling the RUSLE, climate models, and terrain information derived by drones to assess regional and local rain erosion risks for built heritage sites. Consequently, we propose a methodology, exemplifying it in a case study located in northern Ecuador, where the Pambamarca Complex is found. This consist of 18 concentric *Pucaras* located along the Pambamarca volcano. Therefore, we first describe the archaeological relevance of this complex, also focusing on its climate and landscape features. In the following, we describe the assumptions and the derivation of the RUSLE inputs and their calculation using historical and future climate scenarios. Then, we explain how we identified *Pucaras* at high risk of rain erosion to focus on our UAV flight campaigns to identify gully erosion-prone areas. Finally, the advantages and limitations of the proposed methodology are discussed, recommending management actions to conserve the built heritage in the context of climate change and pluvial erosion in the Andes.

## 2. Study area

### 2.1 The Pambamarca complex in northern Ecuador

**Fig 1:**
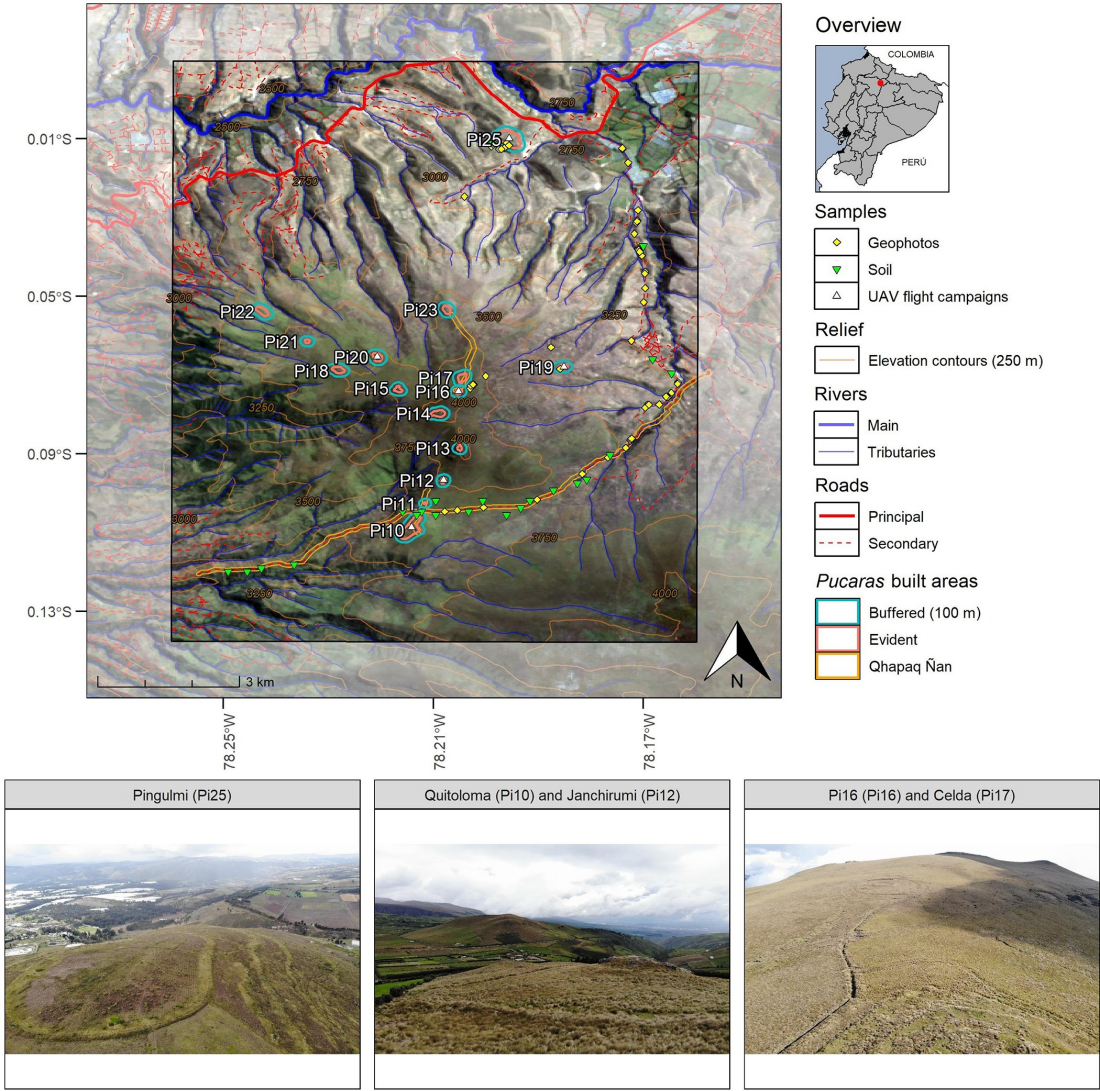
Study area where the Pambamarca volcano and its *Pucaras* are located (background image corresponds to Landsat composite 1986–2000). Bottom: UAV photos provide a visual impression of the *Pucaras*. Map data sources provided by IGM [27].

The Pambamarca Complex is located in Cayambe canton, which is around 35 km from Quito in the northeast of Pichincha province in Ecuador. This region is characterized by an eroded stratovolcano that is 4062 masl called Pambamarca. On the Pambamarca 18 pre-Columbian hilltop fortresses, commonly referred to as *Pucaras*, are found. These installations are the evidence of military preparedness and conflict of local tribes and chiefdoms, collectively called the “País Caranqui”, against the Inka Empire [26]. They are placed following the north-south ridge of the Pambamarca volcano, with distances varying between them varying from 0.1 to 1.7 km and altitudes ranging from 2813 to 4062 masl (Fig 1). Due to this volcanic origin, obsidian artifacts are frequently discovered [28]. Most of the sites were determined to represent the Late Integration Period (AD 950–1530) [29,30], and they are crossed by the Andean roadway system Qhapaq Ñan, declared as part of the UNESCO World Heritage List in 2014 [31]. The Pambamarca Archaelogy Project has conducted several surveys in this region, identifying three types of *Pucaras* and their interior built areas according to Connell et al. [32]. To analyze these installations, we defined a study area in which 15 *Pucaras* were distributed, discarding the *Pucaras* Cangahua and El Quinche (Pi26) because they were not located in our study area and also Pucarito (Pi24) because it was not found during our field work. As their interior built area was not easily identified, we digitized their evident built area on-screen using Google Earth and Bing Maps imagery [33,34]. To ensure also their vicinity with a buffer of 100 m. Details of these areas are also shown in the last columns of Table 1.

**Table 1:** Description of the main characteristics of the *Pucaras* in the study area.

Type	Description	*Pucaras*		Altitude (masl)	Built area (ha)		
		Names	Code				
					Interior	Evident^a^	Buffered^b^
**I**	Constitute large installations with defensive features, such as concentric stone walls (2–5 m height), deep fosses, trenches, and parapets. All installations are constructed from masonry ones.	Quitoloma	Pi10	3800	10.9	14.3	35.1
		Pambamarca	Pi14	4075	10.2	4.3	16.0
		Celda	Pi17	3892	6.6	3.6	14.3
		Tablarumi	Pi18	3791	3.8	3.5	13.8
		Pucara	Pi19	3480	5.3	1.9	10.3
		Censo	Pi20	3796	4.9	3.5	13.7
		Achupallas	Pi22	3400	5.7	3.8	15.0
		Campana	Pi23	3614	6.2	2.8	12.4
		Cangahua^c^	-	4178	-	-	-
**II**	Constitute enclosures, smaller than Type I installations. These are characterized by low stone walls (<2 m height) surrounding central spaces, which presumably served as ceremonial and/or economic centers within the Pambamarca Complex.	El Sombrero	Pi11	3720	0.5	0.2	5.1
		Janchirumi	Pi12	3800	2.5	1.2	8.4
		Jambimachi	Pi13	4078	1.5	1.1	8.4
		Piñan	Pi15	3896	2.8	1.9	10.5
		Pi16	Pi16	3930	3.1	1.7	9.5
		Chiripamba	Pi21	3600	1.0	0.7	7.0
**III**	Constitute fortresses, larger than type I and II installations but highly affected by modern farming. They are characterized by high terrace walls, built with large shaped blocks made by hard-packed consolidated volcanic ash known as *cangagua*. They show no visible standing architecture and occupational debris can be easily found.	Pucarito^c^	Pi24	3020	9.6	-	-
		Pingulmí	Pi25	2997	12.4	16.6	35.8
		El Quinche^c^	Pi26	2813	-	-	-

^a^ Derived from high-resolution imagery

^b^ It constitute a buffer of 100 m from the evident built area.

^c^ Not analyzed in this study.

### 2.2 Climate and landscape overview

Ecuador lies on the Equator, and thus most of its territory experiences a humid tropical climate, except the region crossed by the Andes mountain range where our study area is located. Here, two general climate types can be differentiated according to Pourrut et al. [35]. The Cold Equatorial Climate of High Mountains is prevalent above 3.000 masl. Here, the mean annual temperature fluctuates around 4–8°C, with daily ranges up to 20°C [36]. The annual rainfall oscillates between 800 and 2000 mm, and humidity is almost always above 80%. The second one is found at altitudes below 3000 masl and is named Equatorial Mesothermal Climate, Semi-Humid to Humid. Here, the mean annual temperature fluctuates between 12 and 20°C but rarely decreases below 0°C or exceeds 30°C. The annual rainfall range is 500–2000 mm, and relative humidity between 65 to 85%. Other climate investigations, such as the Köppen-Geiger climate classification [37], indicates for a present scenario (1980–2016) and 80% confidence the following climate types as dominant: tundra (ET), temperate oceanic climate (Cfb) and warm-summer Mediterranean climate (Csb), with surface percentages of 54, 22 and 15% in the study area. However, in a future scenario (2071–2100) with high greenhouse gas emissions (RCP8.5), an expansion of the Cfb (33%) and Csb (22%) types are described at the expense of the ET type (-51%), but with 76% confidence. Furthermore, the Pambarmarca Complex is characterized by a mountainous relief, composed of a volcanic building and cinder cones, surrounded by folded sedimentary rocks, and depositional landforms of little slope (or glacis). Gorges are located on the northern section of the study area, as a result of fluvial erosion of the Pisque River and its tributaries. According to land cover for the year 2014 [38], most of these landscapes are intervened (59.6%), principally by grasslands (31.8%), croplands (21.4%), forest plantations (4.4%) and infrastructures, such as greenhouses (1.7%). The remaining land cover (40.3%) corresponds to plant communities such as the *Páramo* grasslands (11.2%) [39], which dominate elevations above 3600 masl. These are characterized by tufted grasses larger than 50 cm, with peatland soils commonly with C stocks greater than 1500 Mg ha^-1^ and having exceptional water regulation capacity [40,41]. Unfortunately, frequent fires, overgrazing and afforestation with *Pinus* sp. are altering its ecological functions [42]. At altitudes below 3600 masl, and limited to the steep slopes on the western side of the Pambamarca volcano, the High Montane Evergreen Forest (4.7%) [43] is observed. This is a forest that is 10–15 m height, characterized by thick trunks, sometimes twisted and with adventitious roots. They also have shallow (20 to 50 cm) Inceptisols and desaturated-perhydrated Andosols, with a loam to loamy texture, good drainage and the presence of very humiferous soils. The rest of the study area is characterized by the Northern Semi-deciduous Forest and Shrubland of the Valleys (24.2%) [44]. This forest height is around 8–12 m, with a dense undergrowth composed of shrubs, succulent and cactuses. Here, it is common to observe beds of hard volcanic ash known in Ecuador as *cangahua* (or sterile lands), which usually appear on the surface or sometimes buried at shallow depths. Their structure does not easily transmit water and does not permit the penetration of roots, so when it rains heavily, runs off leads to erosion [45]. These areas are not suitable for agriculture and their rehabilitation is costly, requiring at least 8 and 10 hours of bulldozer use to decompact a hectare of land [46].

## 3. Material and methods

To develop our methodology, we first describe data and their processing to derive the needed inputs for the RUSLE. After this, we describe the calculation of the RUSLE inputs using the historical and future climate scenarios to identify *Pucaras* at risk due to rain erosion. Finally, we describe the high resolution DTMs derivation from the UAV flight campaign to identify gully erosion-prone areas.

### 3.1 Rainfall factor (R-factor) for historic and climate change scenarios

We use historical rainfall from the WorldClim dataset [47]. According to the authors, the historic rainfall data was obtained by interpolating selected weather stations and covariates from satellite sensors, achieving an overall correlation coefficient of 0.86 between estimated and observed values. We downloaded a dataset, available for the period 1970–2000 at a spatial resolution of 2.5° (or ~4.6 km), extracted our study area, and downwscaled it to 100 m (i.e. the operational resolution used for RUSLE calculation), following recommendations of Rojas et al. [48]. For this task, we used the random forest [49] algorithm to predict rainfall for the higher resolution using elevation and slope derived from the Shuttle Radar Topography Mission digital elevation model [SRTM-DEM; 50]. We also used the Normalized Difference Vegetation Index [NDVI; 51] and Normalized Difference Built-up Index [NDBI; 52] composited for the period 1986–2000 using the Landsat archive (See Section 3.4). All these covariates are considered appropriate for downscaling climate models [53,54], so we applied the next processing steps to predict rainfall at higher resolution:

1. Dissagregate the rainfall raster to 100 m resolution and convert it into spatial points.
2. Extract covariates information to each spatial point.
3. Random sample spatial points in training (20%) and validation (80%). This ratio follows Sulaiman et.al [55] for rainfall prediction.
4. Train the random forest algorithm, using the R library “randomForest” [56] and its default calibration parameters, i.e. 500 for the number of trees and the square root number of randomly sampled variables as candidates in each split (mtry).
5. Predict the downscaled rainfall raster and derive its accuracy metrics (e.g. RMSE and R-squared) using the validation set.

After applying the above procedure, the R-squared of the downscaled historical rainfall model was 0.85 (Table 2). To verify that the rainfall value ranges were consistent with those described in Section 2.2, we split the study area into two regions applying a threshold of 3000 masl. We observed that, above it, values averaged 906 mm (*SD* = 38), while below it, these averaged 804 mm (*SD* = 10). This met our expectations, i.e., elevated areas were wetter than those at lower elevations. Following this, we downloaded climate change scenarios from the CMIP Phase 6 [57] for four modeling centers. It was preferred to work with several climate models as there is no absolute certainty of climate change impacts in mountainous areas, specially for local scales [58]. The four modeling centers were chosen because they demonstrated the best performance for South America after the assessment of Cannon [59]. These models included four time periods covering the 21th century (2021–2040, 2041–2060, 2061–2080, and 2081–2100) for four Shared Socio-economic Pathways (SSP) or greenhouse emission scenarios, which can be described as follows:

- SSP1-2.6, the green growth scenario with a warming range of 1.3–2.9°C. It assumes efficient resource use, a preference for sustainable production, less land use and lower anthropogenic greenhouse gas emissions at the end of the 21th century [60].
- SSP2-4.5, the middle-of-the-road scenario with a warming range of 2.1–4.3°C. It assumes unevenly development and income growth, a slow decrease in fossil fuel dependency and energy use and moderate population growth until the second half of the 21th century [61].
- SSP3-7.0, the regional rivalry scenario with a warming range of 3.0–6.2°C. It assumes high greenhouse gas emissions, low mitigation capacity and decreased forest area with a large expansion of cropland and pasture land [62].
- SSP5-8.5, the worst-case scenario with a warming range of 3.8–7.4°C. It assumes a rapid economic growth, very high fossil fuel use, double global food demand and tripled energy demand and greenhouse gas emissions [63].

**Table 2:** Climate change scenarios used in this study, sorted by performance scores for South America according Cannon [59].

Modelling center	Model name	Grid size (°)	Average				Reference
			R-squared^a^	Modelerror^b^	Annual rainfall (mm)^c^	R-factor (MJ*mm*ha^-1^*h^-1^*y^-1^)^c^	
WorldClim	Historical	2.5	0.85	-	876.4	345.4	[47]
Institut Pierre Simon Laplace	IPSL-CM6A-LR	1.98	0.84	0.50	1219.3	465.4	[65]
JAMSTEC, AORI, NIES, and R-CCS	MIROC6	1.41	0.82	0.55	907.4	356.2	[66]
Centre National de Recherches Meteorologiques and Centre Europeen de Recherche et de Formation Avancee	CNRM-CM6-1	1.41	0.82	0.85	1073	414.1	[67]
Geophysical Fluid Dynamics Laboratory	GFDL-ESM4	1.00	0.83	0.95	738.1	296.9	[68]

^a^Averaged for all downscaled climatic scenarios except for the historical scenario

^b^Averaged from four error coefficients (0–2), which represent the classification performance achieved by four atmospheric circulation systems described by Cannon [59]

^c^Averages derived for all the study area. For Worldclim, it was calculated for the period 1970–2000, while for the rest of the models, averages were derived for the SSP5 scenario, period 2080–2100.

In total, we acquired 16 datasets for each modeling center, except for GFDL-ESM4, for which we acquired 12 as it does not include SSP2-4.5. To prepare them, we applied the same procedure as that for the historical rainfall dataset, i.e., extract the study area and downscale it to 100 m. As a result, all downscaled models achieved R-squares above 0.8 (Table 2). With these data, we computed the R-factor, which is defined as an index of the erosion force of rainfall. We applied the model described in Merritt et al. [64] as:

R=38.5+0.35*P (1)

Where *R* represent the R-factor and *P* is the total annual precipitation in mm. We applied equation mentioned above to all datasets to obtain a total of 65 rainfall factors for the SSP scenarios and models selected. Output values are in MJ*mm*ha^-1^*h^-1^*yr^-1^ and varied by location, but also among the SSP scenarios. Table 2 shows a summary of these values.

### 3.2 Soil erodibility factor (K-factor)

The K-factor represents the susceptibility of a soil to erode, expressed in properties such as organic matter content, soil texture, soil structure and permeability. To apply it, we first downloaded soil texture data from SoilGrids [69]. This is a system of global digital soil mapping derived from soil profiles and different environmental covariates (e.g. climate, land cover, terrain morphology) using machine learning at a spatial resolution of 250 m. According to its source, 96 soil profiles were observed in Ecuador for predict soil features at six depth intervals (0–2 m). We downloaded the profile for the top soil layer (0–5 cm) as is considered the most affect by erosion by water [70], and for four features: sand, silt, clay and organic carbon percentage content. We downscaled them to 100 m with the method and covariates described in Section 3.1, as they are also observed to predict soil textures [71]. Despite the fact they performed worse than those from rainfall, their R-squared was greater than 0.6 in all cases, except for the silt texture which achieved 0.51. To evaluate if they agree with the soil description of Section 2.2, we also divided the study area in two regions but now applying a threshold of 3600 masl to isolate the *Páramo* grasslands. As we expected, the latter achieved a higher average organic content (*M* = 53.7, *SD* = 0.8%) than those areas observed at lower altitudes (*M* = 49.0, *SD* = 3.2%). After this, we proceeded with the K-factor calculation, applying the equation of Williams [72], which requires the percentage composition of soil particles and organic carbon. Its equation is:

$$K=0.1317*f_{csand}*f_{cl-si}*f_{orgc}*f_{hisand}$$ (2)

Where *K* is the K-factor, *f*_*csand*_ is a variable that decreases *K* when high coarse-sand content occurs or increases it when little sand occurs; *f*_*cl*−*si*_ is a variable that decreases *K* when the ratio between clay and silt is high; *f*_*orgc*_ is a variable that decreases *K* when high organic content is present; and *f*_*hisand*_ is a variable that decreases *K* when soils have a very high sand content. These variables were further derived with the following equations:

$$f_{csand}=\left(0.2+0.3*exp[-0.0256*p_{sand}*(1-\frac{p_{silt}}{100})]\right)$$ (3)

$$f_{cl-si}={\left(\frac{p_{silt}}{p_{clay}+p_{silt}}\right)}^{0.3}$$ (4)

$$f_{orgc}=\left(1-\frac{0.25*P_{org}}{P_{org}+exp[3.72-2.95*P_{org}]}\right)$$ (5)

$$f_{hisand}=\left(1-\frac{0.7*(1-\frac{p_{sand}}{100})}{\left(1-\frac{p_{sand}}{100})+exp[-5.51+22.9*(1-\frac{p_{sand}}{100})\right]}\right)$$ (6)

where *p*_*sand*_ is the sand fraction content (0.05–2.00 mm diameter) in percentage; *p*_*silt*_ is the silt fraction content (0.002–0.05 mm diameter) in percentage; *p*_*clay*_ is the clay fraction content (<0.002 mm diameter) in percentage; and *p*_*org*_ is the organic carbon content in percentage. After these equations were calculated, the K-factor is expressed in ton*ha^-1^MJ^-1^*mm^-1^. We observed that it averaged 0.029 (*SD* = 0.0006), ranging from 0.024 to 0.03 in our study area.

### 3.3 Slope length (S-factor) and steepness factors (L-factor)

The next step in the methodology was the S- and L-factors calculation. The latter determines the impact of slope length due to the distance between the point of origin of overland flow and the deposition point (or channel). Furthermore, the S-factor accounts for the effect of slope steepness, being higher when the slope is steeper [73,74]. Following the approach of Desmet and Govers [75], the L-factor can be derived through:

$$L_{i,j}=\frac{{\left(A_{i,j-\mathrm{in}}+D^{2}\right)}^{m+1}-A_{i,j-\mathrm{in}}^{m+1}}{D^{m+2}*x_{i,j}^{m}*22.13^{m}}$$ (7)

$$m=\frac{\beta }{\left(1-\beta \right)}$$ (8)

$$\beta =\frac{\left(sin\frac{\Theta }{0.0896}\right)}{\left[0.56+3*{\left(sen\Theta \right)}^{0.8}\right]}$$ (9)

where *L*_*i*,*j*_ is the impact of slope length factor for cell (*i*,*j*); *A*_*i*,*j*−in_ is the contributing area at the inlet of cell (*i*,*j*) measured in m^2^; *D* is the cell size in m; *X*_*i*,*j*_ = *sinα*_*i*,*j*_+*cosα*_*i*,*j*_; *α*_*i*,*j*_ is the aspect direction of cell (*i*,*j*); *m* is the ratio *β* of the rill to interill erosion; and *Θ* is the slope angle in degrees. To complete the S-factor, the following equations are applied:

S=10.8*sinΘ+0.03,Θ<0.09 (10)

S=16.8*sinΘ−0.5,Θ≥0.09 (11)

where *S* is the S-factor. To derive them, we used the System for Automated Geostatistical Analysis [SAGA-GIS; 76] to first fill surface depressions of the SRTM-DEM (See Section 3.1) with the function “Preprocessing—Fill sinks (Wang & Lui)” [77] to force areas to flow downstream where pooling occurs. Then, we applied the function “Hydrology—LS factor, Field Based” [78], which outputs the multiplication of S- and L-factors. Despite this factor is dimensionless, we obtained an average of 11 (*SD* = 21) for all of the study area, where it averaged 7.6 (*SD* = 10.9) on the higher slopes (>25°), and 12.4 (*SD* = 22.4) on the lower slopes (<25°).

### 3.4 Land cover management factor (C-factor)

The ratio of soil loss from land cover under a specific vegetation canopy or crop management system is defined as the C-factor. It implies that, holding other factors constant, dense canopies (e.g., forests) reduce soil erosion better than bare soil or sparse vegetation. Varying from 0 to 1, higher C-factor values correspond to more erodible areas. Its calculation can be performed using specific land use-land cover type coefficients obtained from the literature [79]. Nevertheless, other approaches using remote sensing data are useful, specially when C-factor coefficients are not available for specific land use-land cover types (e.g. *Páramo* grasslands). For this reason, we followed Durigon et al. [80] and applied the following equation:

$$C_{rA}=0.1*\left(\frac{-NDVI+1}{2}\right)$$ (12)

$$NDVI=\left(\frac{NIR-RED}{NIR+RED}\right)$$ (13)

where *C*_*rA*_ is the land cover management factor, *NDVI* is the Normalized Diference Vegetation Index, *RED* is the surface spectral reflectance in the red band, and *NIR* is the surface spectral reflectance in the infrared band. The NDVI is a well-known spectral index, which is widely used to highlight green vegetation vigor, differencing the spectral reflectance between the red and infrared bands of a multispectral satellite image. To calculate the C-factor, we used Google Earth Engine [81] and created a routine to use the U.S. Geological Survey Landsat Surface Reflectance Tier 1 product. This dataset measures the fraction of incoming solar radiation that is reflected from Earth’s surface to the satellite sensor removing the atmospheric effect which can negatively impact it [82]. To process it in Google Earth Engine, we applied the next steps:

1. Define a geometry to extract the study area from the Landsat 4, 5, and 7 Surface Reflectance Tier 1 product.
2. Filter images by band, specifying the blue (B1), green (B2), red (B3), infrarred (B4) and shortwave infrarred (B5) bands.
3. Filter images by date, specifying the period from 1986-07-13 to 2000-09-29 and the images acquired during the dry season (June–September).
4. Apply the pixel quality bitmask and remove the pixels classified as water, clouds, clouds shadow, and snow.
5. Calculate the median for the whole period and for the image bands. Then, calculate the NDVI and NDBI (see sections 3.2 and 3.3).
6. Aggregate the output pixel size for NDVI and NDBI at 100 m but keep it at 30 m in bands 1, 2, and 3 to derive a natural color composite for mapping purposes (See Fig1).

In total, 60 images were available for the period mentioned above and were used in the median composite to obtain the NDVI, whose mean was 0.44 (*SD* = 0.12) for the entire study area. We calculate the C-factor applying Eq 12. We observed that values of the C-factor averaged 0.16 (*SD* = 0.16) at forested areas; 0.25 (*SD* = 0.15) in croplands; and 0.25 (*SD* = 0.17) in grasslands and shrublands. All these values are dimensionless and those related to cropland coincided with those described by Benavidez et al. [83].

### 3.5 Operation of RUSLE and validation of its results with the historical climate model

**Fig 2:**
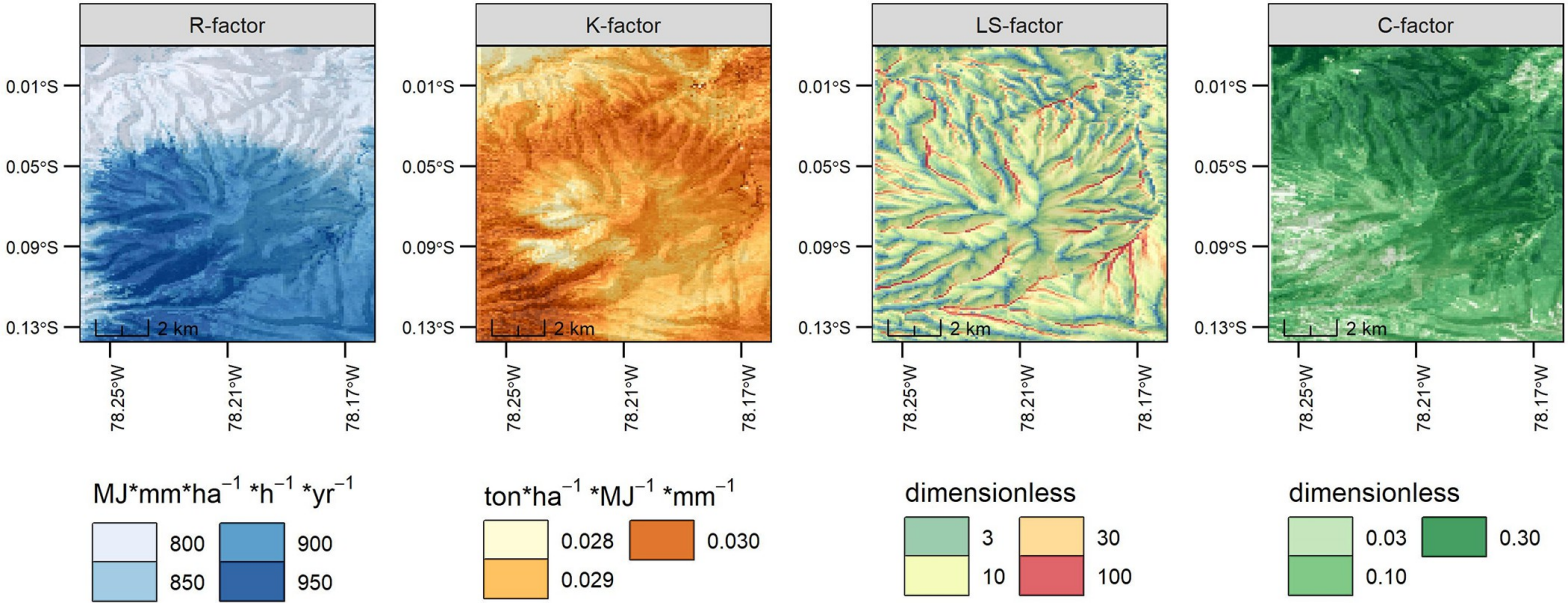
Spatial representation of the factors required in the RUSLE calculation.

After calculating the necessary factors (Fig 2), all of them at 100 m, we applied the RUSLE equation, which is described as

A=R*K*L*S*C*P (14)

where *A* is mean annual soil loss, *R* is the R-factor, *K* is the K-factor, *L* is the L-factor, *S* is the S-factor, *C* is the C-factor and *P* is the support practice factor (P-factor). The predicted mean annual soil loss is expressed in ton*ha^-1^*yr^-1^, but *P* is rarely included in the calculation as it requires extensive knowledge of the study area management actions [84] and it is not straightforward to predict in the context of climate change [85].

Because of this, we assigned a value of 1 such that it has no effect on the calculations. As we included four climate models for its application to the RUSLE, we first calculated it using the R-factor obtained with the historical climatic model. Then, we replace it with the one produced with each of the climate change models *M* = {*CNRM*−*CM*6−1, *GFDL*−*ESM*4, *IPSL*−*CM*6*A*−*LR*, *MIROC*6}, their emission scenarios *SPP* = {1−2.6; 2−4.5; 3−7.0; 5−8.5}, and their time steps *T* = {2021−2040, 2041−2060, 2061−2080, 2081−2100} in the RUSLE. This can be represented as

$$A_{hist}=R_{hist}*K*L*S*C$$ (15)

$$A_{scn}=R_{\left(M,SPP,T\right)}*K*L*S*C$$ (16)

where *A*_*hist*_ is the mean annual soil loss derived with the historical rainfall R-factor *R*_*hist*_; and *A*_*scn*_ is that derived from the future rainfall R-factor *R*_(*M*,*SPP*,*T*)_, considering a specific *M*, *SPP*, and *T* scenario. After these calculations, we subtracted *A*_*hist*_ from *A*_*scn*_ to quantify their increase or decrease. This can be expressed as

$$A_{diff}=A_{scn}-A_{hist}$$ (17)

where *A*_*diff*_ is the increase/decrease in the mean annual soil loss induced by a specific future climate model. This measure allowed us to quantify their impacts and identify which scenarios can be qualified as the most extreme. Nevertheless, as *A*_*hist*_ is crucial for quantifying differences, we first validated its results. For this, we proceed according to two approaches. The first applied a qualitative procedure, inspired by the work of Bosco et al. [86] and Marondedze and Schütt [87] which focuses on the identification of erosion signs at different sites of the RUSLE model. We then collected 11402 geophotos using mobile cameras and a GoPro Hero 7 camera mounted on the roof of a vehicle (photographs were acquired in automatic mode, every two seconds). As their number was high, we filtered those showing a soil profile or erosion scars to interpret them based on the soil erosion categories described by Warren et al. [88] (Table 3).

**Table 3:** Soil erosion categories used for visualization and field validation of RUSLE, after Warren et al. [88].

Soil erosion by water (ton*ha^-1^*yr^-1^)	Area (ha)	Samples (count)		Erosion categories	Description
		Soil	Geophotos		
0–5	3694	9	20	Low	* Few signs of water movement. * Minimal pedestaling of plant crowns. * Some signs of scouring, litter dams and surface stones may be present.
5–10	3943	4			
10–20	4726	3	20	Medium	* Runoff patterns and small rills may be evident. * Some pedestaling of plant crowns. * Scouring, litter dams, and surface stones generally evident. * Density and vigor of plants may be lower than uneroded areas due loss of soil fertility. * Surface may appear marginally rockier or gravellier textured than uneroded areas due to erosion of fine soil particles.
20–100	5006	4	20	High	* Runoff patterns evident. Significant rills and gullies often present. * Evident pedestaling of plant crowns. * Scouring, litter dams, and surface stones evident. * Density and vigor of plants often lower than uneroded areas due to loss of soil fertility. * Species composition likely to include more weeds than uneroded areas due to loss of soil fertility, exposure of subsoils and importation of seeds via overland flow of water. * Surface may appear rockier or heavier textured than uneroded areas due to loss of fine soil particles.
> 100	887	3			

We sampled 20 for each category to evaluate *A*_*hist*_, taking into account their distribution in our study area (See Fig 1). We have computed a confusion matrix comparing the mean annual soil loss value in RUSLE (prediction) and the corresponding erosion values interpreted from the geophotos (reference). Given the next matrix as example, we computed the next metrics

**Table:** 

	Reference	
Prediction	No	Yes
No	*TN*	*FP*
Yes	*FN*	*TP*


Sensitivity=TP/(TP+FN) (18)

Specificity=TN/(TN+FP) (19)

where *TN* are the true negatives; *FP* are the false positives; *FN* are the false negatives and *TP* are the true positives. These are used to derive *Sensitivity* and *Specificity* which refer to the rate of true positives and true negatives, respectively. Next, we calculated the *OverallAccuracy* which can be defined as the weighted average of the sensitivity and specificity as

OverallAccuracy=(TN+TP/TN+FP+FN+TP) (20)

A final accurancy metric calculated was the *Kappa*, which is an index to measure the agreement of two categorical scales by applying the following equations:

$$P_{o}=(TN+TP/TN+FP+FN+TP)$$ (21)

$$P_{correct}=(TN+FP/TN+FP+FN+TP)*(TN+FN/TN+FP+FN+TP)$$ (22)

$$P_{incorrect}=(FN+TP/TN+FP+FN+TP)*(FP+TP/TN+FP+FN+TP)$$ (23)

$$P_{e}=P_{correct}+P_{incorrect}$$ (24)

$$Kappa=P_{o}-P_{e}/1-P_{e}$$ (25)

where *P*_*o*_ is the probability of agreement, and *P*_*e*_ is the probability of random agreement. The second approach was based in a quantitative procedure. This consisted of collecting soil samples throughout the study area by applying a stratified sampling to six soil erosion categories in *A*_*hist*_. These categories correspond to the *A*_*hist*_ ranges 0–5, 5–10, 10–20, 20–100 and >100. For each stratum, at least three samples were collected. The samples consisted of soil matter (~1 kg) collected below the top soil layer (10 cm) at the sample site. These samples were then taken to an independent laboratory for texture analysis. In total, we collected 23 samples in the study area (See Fig 1), and to corroborate that *A*_*hist*_ predicted accordingly, we expected that increases of clay and silt fractions correlate negatively with *A*_*hist*_, as such soil textures are easily transportable [89]. By contrary, sand fraction must correlate positively with *A*_*hist*_ as result of soil splash and wash erosion of clay and silt particles. For quantifying this similitude, we computed the Spearman correlation metric as it is is less sensitive to outliers than Pearson. After validation, we reported the results of the *A*_*hist*_ extracted and averaged its values according to the buffered area of *Pucaras*. This is shown later in Section 4.1.

### 3.6 Priorization of Pucaras and identification of gully erosion

Following, we prioritized *Pucaras* according to their rain erosion exposure. For this, we used their buffered areas and averaged *A*_*diff*_ values for each model, SSP and time step. We ranked *Pucaras* based on these averages and separated those occurring in the first three positions to identify only the most affected in each future climate model. Then, we identified the most frequent and selected six of them (See later in Section 4.3). We chose this number to have a reasonable number of UAV flight campaigns, given that some *Pucaras* were located in areas of difficult to access. To gain access to the *Pucaras* areas, we followed the Heritage Law of Ecuador [90] and contacted the National Institute of Cultural Heritage, i.e., the national authority in charge of research, conservation and restoration of heritage properties in Ecuador. We also contacted to the local goverment in our study area, i.e., the Municipality of Cayambe (GADIP). Since we did not conduct excavations, collect archaeological remains, endangered or protected species, we were informed that we did not need permission given the non-intrusive methodology of this research; however, it was suggested that to ask community leaders for verbal or written permission to conduct flights. Following the latter, we first consulted with the people present at the *Pucara* sites when we were unable to contact community leaders but also held a workshop with the Pambamarca community to socialize our research. For our flight campaigns, we used a DJI Mavic Air drone, which is agile, relatively inexpensive (<USD 1k) and lightweight (430 gm). It is equipped with a 35 mm lens, capable of taking pictures in red, green and blue (RGB) with a size of 4056*3040 pixels and a resolution of 72 dots per inch (DPI) in high resolution (HD) mode. To control it, we used the DJI GO 4 v4.3.37 app [91] and two flight batteries (they last approximately 21 minutes). As the *Pucaras* areas presented harsh conditions for flights (e.g., unexpected high winds or rains, complicated terrain for take-off and landing), we preferred the manual mode to control the drone. In all cases, we flew at 100 m from the take-off site to optimize battery time as some *Pucara* areas were large, but also to capture enough detail in the photographs. To maximize their overlap, we set the trigger in automatic mode to the minimum time supported (i.e., two seconds). We followed the next procedure on each of the *Pucara* flight campaings:

1. Set the drone camera to automatic mode for white color balance and camera sensitivity to light (ISO). Orient the camera gimbal to ~-15° (oblique).
2. Take-off from the approximate center of the *Pucara*, elevate it to ~20 m and take oblique photographs. Orient the camera gimbal to ~-90° (vertical).
3. Further elevate the drone until it rises ~100 m from the take-off site and proceed to navigate in a grid path fashion until the area of the *Pucara* is completed.
4. Land the drone with hand catch to avoid collision.
5. Repeat the flight if the *Pucara* area is not captured.

**Fig 3:**
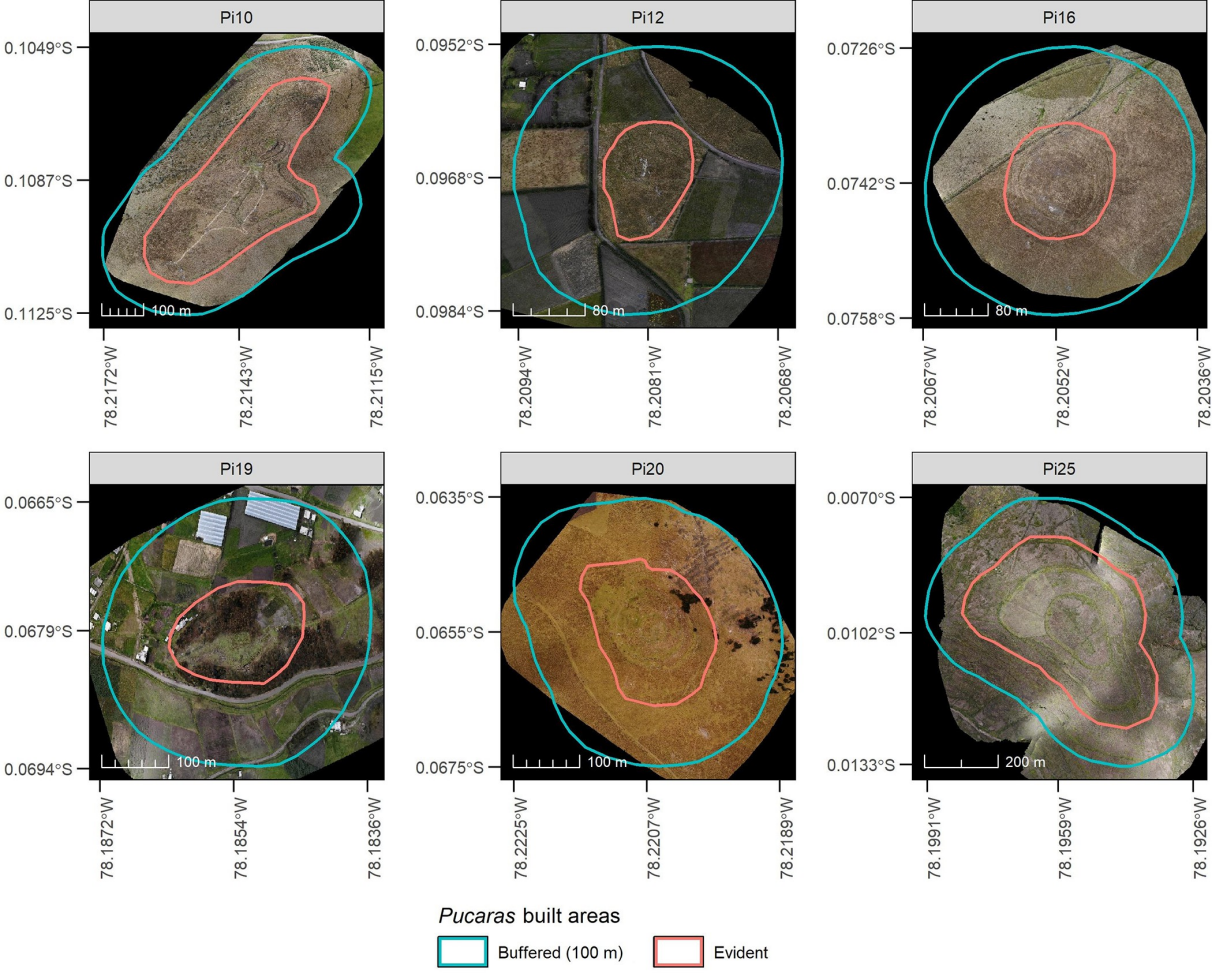
*Pucaras* orthophotos derived from the UAV flights campaigns.

In total, we collected 1698 RGB photographs in five flight campaigns, and depending on the *Pucara* size, this number ranged from 160 to 636 images. After flight campaigns were completed, we separate oblique photographs from those acquired with the camera gimbal in vertical mode and at ~100 m. After this step, we used OpenDroneMap, an open-source command line toolkit for processing aerial drone imagery [92]. We used its web application called WebODM and applied the default parameters described in the latter refence for data processing, except for some recommended ones: 1024 pixels for the depthmap resolution, and Brown-Conrady as camera model [93]. We computed DTMs, as they highlight the bare earth and are recommended to represent erosion effects when surface vegetation is degraded or removed [94]. The output DTMs’ spatial resolution ranged between 13.4 to 16.5 cm and their accuracy are summarized in Table 4. The horizontal-vertical absolute accuracies averaged 1.6–4.1 m, while their relative (i.e., independent of the real-world position) averaged 0.3–0.55 m. Since our objective was not a high-precision topographic survey, but to obtain a terrain model suitable for identify gully erosion areas, we considered that the relative accuracy achieved was sufficient for our work. In addition, we also derived orthophotos to visualize land cover and *Pucaras* spatial context. Following these steps, we obtained the stream power index, which is a predictor of ephemeral gullies [95] and is recommended for identifying locations where soil conservation measures are required to reduce the erosive effects of concentrated surface runoff [96]. This index is calculated with the following equation:

SP=ln(DA*tan(G)) (26)

where *SP* is the stream power index, *DA* is the upstream drainage area and *G* is the slope in radians. To calculate it, we also used the SAGA-GIS software by first filling surface depressions in the high resolution DTM (See Section 3.3). Then, we applied the function “Compound Analysis—Basic Terrain Analysis” to derive inputs of Eq 18. These allowed to execute the “Hydrology—Stream Power Index” function, which derives *SP* in watt*m^-2^. To better interpret it, we reclassified it into 20–30 and >30 ranges, considering that they are related to erosion-dominated areas [97]. To report these results, we derived maps and calculated erosion-dominated areas from evident built area of *Pucaras*. This was carried out because the buffer area was not fully captured in all flight campaigns due to its extension (Fig 3). In addition, to improve the visualization of altitudinal gradients, we calculated the hull of the ellipsoid [98] from the evident built areas of *Pucaras* and then derived the long axis and short axis. These two axes were used to extract DTMs values every 0.5 m, highlight *Pucaras* terraces and plot elevation profiles. All these calculations that were described throughout Section 3 without a specific software or application reference, were performed in the R language [99] using libraries such as sf [100], ggplot2 [101], caret [102], cluster [103], raster [104], mefa [105], reshape [106], data.table [107], and flexpolyline [108]. The erosion calculations also used information and code provided by Herrnegger and Schürz [109].

**Table 4:** Flight campaigns description and summary of the obtained DTMs.

*Pucara* (code)	Acquisition date(s)	Relative height (m)		Images (count)	Coverage (ha)	DTM resolution (cm)	GPS error (m)	Horizontal-Vertical accuracies (m)	
		Mean	SD					Absolute^a^	Relative^b^
**Pi10**	2022-03-26 2022-04-10	101.9	7.4	385	33.38	15.3	3.8	2.9–6.1	0.1–0.3
**Pi12**	2022-04-10	95.0	11.9	160	5.68	14.5	1.4	0.8–2.3	0.2–0.3
**Pi16**	2022-05-23	98.1	8.4	171	3.93	13.4	1.0	1.1–1.1	0.3–0.7
**Pi19**	2022-05-19	101.3	0.4	208	9.78	16.5	2.2	2.7–3.0	0.7–1.1
**Pi20**	2021-09-12	103.5	0.3	138	8.58	15.4	1.1	0.7–1.9	0.2–0.3
**Pi25**	2022-02-20 2022-05-19	100.0	5.1	636	36.97	15.5	5.1	1.6–10.4	0.3–0.6

^a^ Depend on real-world position

^b^ Do not depend on real-world position.

## 4. Results

### 4.1 Accuracy assessment, and evaluation of RUSLE with the historical climate data

**Fig 4:**
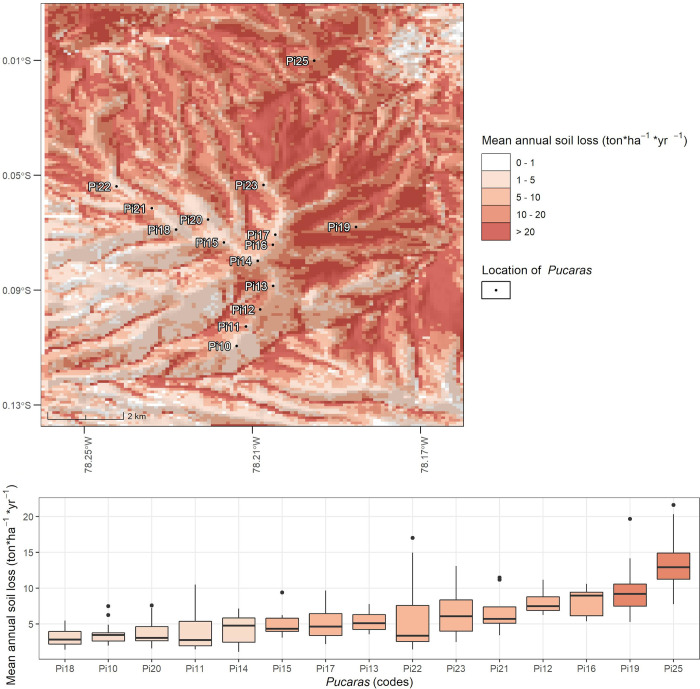
Spatial representation of the mean annual soil loss derived from the historical rainfall R-factor using the RUSLE model. Bottom: Boxplots of values observed in the buffer area of *Pucaras*.

After the RUSLE calculation, we evaluated its plausibility and accuracy with the data collected in the field. Threfore, we first report those derived with the qualitative approach, where geophotos were used. The overall accuracy indicated a satisfactory score of 0.75, i.e., around three samples out of every ten were correct. However, the Kappa index reached a moderate score of 0.62, indicating that the prediction of some classes failed. These are described in Table 5, where sensitivity and specificity metrics are shown. Here, it can be seen that all classes achieved values above 0.8 for these metrics, except for the middle class, which achieved a sensitivity of 0.55. This indicates that the model was more prone to false negative errors, especially for this class, but in a minor proportion for the higher class. Furthermore, the quantitative assessment indicated that clay and silt fraction achieved a negative correlation with RUSLE being these -0.35 and -0.32, respectively. However, both were not statistically significant, as their p-values achieved 0.09 and 0.13, respectively. This was different for the sand fraction which reached a positive correlation of 0.63 with RUSLE and was statistically significant (p-value 0.001). These results confirmed partly our expectations (See Section 3.5) but they were considered reliable enought to continue with our analysis. After evaluating the accuracy, we now report the results of *A*_*hist*_ (or the RUSLE with the historical climate model) in the context of *Pucaras*; therefore, Fig 4 shows its spatial representation. Below it, values in the buffered areas of the *Pucaras* are represented as boxplots, while their colors represent a reclasification of their averages according to the erosion classes described in Table 3. These boxplots are ordered according to their magnitude, amd thus it can be noted that Pi25 and Pi19 lead the ranking with medium erosion (10–20 ton*ha^-1^*yr^-1^). Both are located on the eastern and northeastern slopes of the Pambamarca volcano, covering a lower altitude range from 2974 to 3494 masl. Following these, *Pucaras* Pi16, Pi12, Pi21, Pi23, Pi22, Pi13, Pi17 and Pi15 are characterized by low erosion (5–10 ton*ha^-1^*yr^-1^). They follow the crest of the Pambamarca volcano, located on its central and western slopes, which cover an altitudal range from 3368 to 4025 masl. In the last positions, *Pucaras* Pi14, Pi11, Pi20, Pi10 and Pi18 were also classified with low erosion but with lower *A*_*hist*_ values (0–5 ton*ha^-1^*yr^-1^). They are also located on the central and western slopes but in a higher altitudinal range, between 3655 and 4062 masl (the latter includes the summit of the Pambamarca volcano).

**Table 5:** Confusion matrix for low (0–10 ton*ha^-1^*yr^-1^), medium (10–20 ton*ha^-1^*yr^-1^), and high (>20 ton*ha^-1^*yr^-1^) erosion categories after assesment with geophotos.

		Reference			Specificity
		Low	Medium	High	
**Prediction**	**Low**	18	2	0	0.95
	**Medium**	2	11	4	0.85
	**High**	0	7	16	0.82
**Sensitivity**		0.90	0.55	0.80	

### 4.2 Future climate change and subsequent RUSLE predictions

**Fig 5:**
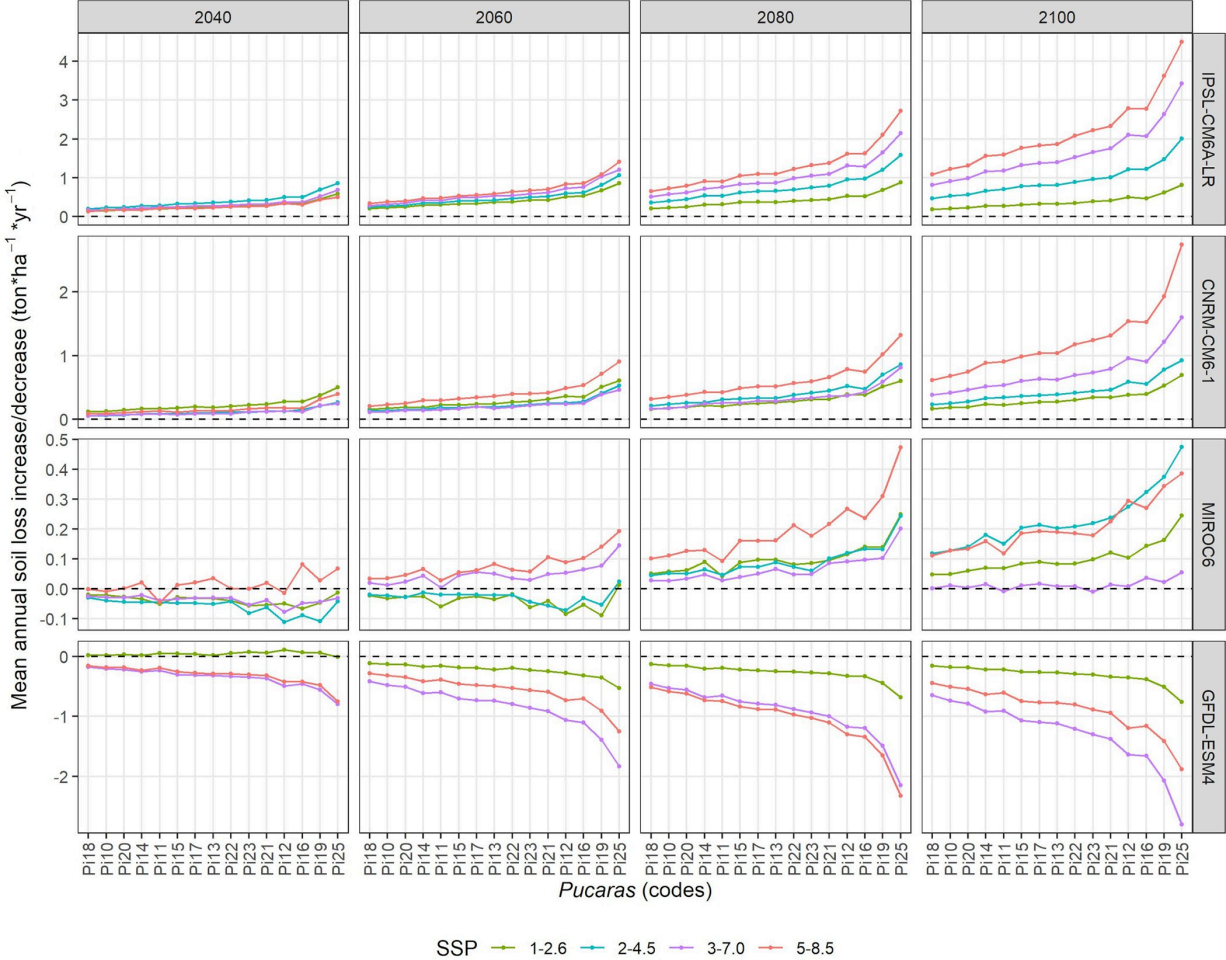
Mean annual soil loss increase/decrease between current and future conditions derived with the climate simulations from the different modeling centers (rows), SSPs (color lines), and time steps (columns) for the *Pucaras* analyzed in this research.

**Fig 6:**
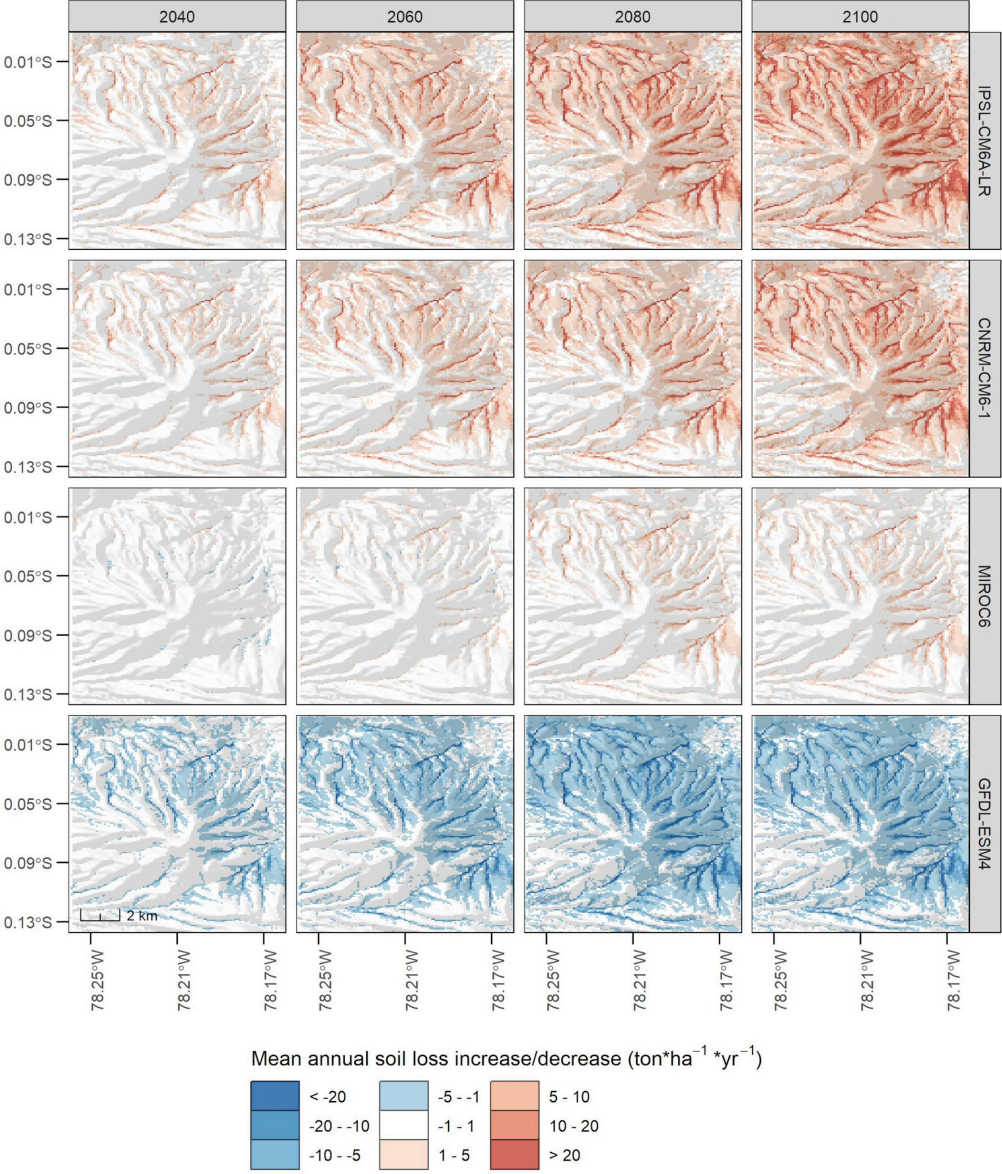
Spatial representation of mean annual soil loss increase/decrease derived with the different modeling centers (rows) and time steps (columns) with the SSP5-8.5.

We now report results of the differences between *A*_*scn*_ and *A*_*hist*_ to highlight their effect on the RUSLE predictions. Since the combinations of modelling centers, SSPs and time steps reached a total of 65, we summarized them in Fig 5 to facilitate their report. Here, the x-axis shows the *Pucara* codes which are ordered according to their average annual soil loss increase/decrease values, shown in the y-axis. We present the SSPs as colored lines but the rest of the parameters as facets in the graph. For this reason, we ranked the facets top-down according to the positive (increase in annual soil loss) or negative (decrease in annual soil loss) effect produced in the RUSLE predictions. Therefore, it can be observed that the IPSL-CM6A-LR model achieved the strongest positive effect, indicating an increase in the mean annual soil loss from 0 to 4 ton*ha^-1^*yr^-1^ for all SSPs and time steps. Similarly, the CNRM-CM6-1 model showed a positive effect but with moderate values (0–2 ton*ha^-1^*yr^-1^). Differently, the MIROC6 model showed a positive effect but with a weak intensity (0–0.5 ton*ha^-1^*yr^-1^) and, in some cases, a negative effect with values from 0 to -0.1 ton*ha^-1^*yr^-1^ for SSPs 1–2.6, 2–4.5 and 3–7.0 during time steps 2040 and 2060. At the far end, the GFDL-ESM4 model indicated the strongest negative effect with moderate values from 0 to -2 ton*ha^-1^*yr^-1^ in all SSPs and time steps. Interestingly, SSP 3–7.0 (regional rivalry) showed a stronger negative effect than SSP 5–8.5 (worst-case), which was somewhat different from what was expected. Furthermore, a spatial representation of the RUSLE models using SSP 5–8.5 is shown in Fig 6. We chose this SSP to more clearly show the negative or positive effect on the RUSLE prediction, as it is the stronger SSP in almost all cases. Then, it can be appreciated that IPSL-CM6A-LR highlights strong increases (5 - >20 ton*ha^-1^*yr^-1^) principally on the eastern (longitude -78.21 - -78.17) and northern (latitude -0.05 - -0.01) slopes on the Pambamarca volcano. Similarly, but with less extensive effect, the CNRM-CM6-1 model better highlights the zones that correspond mainly to riverbeds and gorges in the vicinity of the volcano. With mostly a positive slight effect (1–10 ton*ha^-1^*yr^-1^), the MIROC6 model highlights the latter geoforms. Neverthless, in the time step 2040, a negative effect with mostly weak values (-1 to -5 ton*ha^-1^*yr^-1^) can be appreciated in the eastern areas of the volcano. Finally, the GFDL-ESM4 model showed a similar pattern to the one observed in the CNRM-CM6-1 model, but only with a negative effect. Here riverbeds and gorges seem to drastically reduce their erosion capability, reaching values of around -5 - <20 ton*ha^-1^*yr^-1^. It should be noted that in almost all models and time steps cases, the southwestern slopes of the Pambamarca volcano (latitude -0.013 - -0.05; longitude -78.21 - -78.25) remained with values of around -1 to 1 ton*ha^-1^*yr^-1^. In this respect, these areas correspond to mostly forested areas. Similarly, a small spot at the northeastern section (latitude -0.01; longitude -78.17) is also observed, but in this case, it corresponds to greenhouse infrastructures used for flower production. To report on which *Pucaras* will be most affected by erosion according to these models, we describe a tabulation of these results in the following section.

### 4.3 *Pucara* prioritization according to their rain erosion gain according to the future climate models

As shown on the on the x-axis of Fig 5, *Pucaras* Pi25, Pi19, Pi16, Pi12, and Pi21 ranked in the top positions after ordering their mean annual soil loss increments. However, this graph reorders them based on an overall average to facilitate the drawings, which is different when we rank them based on the frequency of the first three positions of maximum erosion in each model. Table 6 shows a ranking based on these frequencies, whose sum is shown in the last column. It can be observed that Pi19 and Pi25 were ranked at the first three positions 12 times, followed by Pi12 and Pi16 with 6 times. At the bottom end, Pi10, Pi18 and Pi20 ranked only three times. Other *Pucaras* are not present in this ranking since they were in other rank positions from fourth to fifteenth. Moreover, the number of times reached by models was different among *Pucaras*, but IPSL-CM6A-LR, CNRM-CM6-1 and MIROC6 stand out for having the largest proportion (12 times in each case), followed by CNRM-CM6-1 (9 times). To better understand this ranking, Table 7 shows these frequencies according to the SSPs and time steps. Here, it can be seen that almost all *Pucaras* achieved at least one occurrence in the first three positions of all SSPs, except Pi10, Pi18 and Pi20, which did not occur in SSP 2–4.5. On the other hand, Pi19, Pi25, Pi12 and Pi16 indicated that they occurred in the first three positions of the 2080 and 2100 time steps, while Pi10, Pi18 and Pi20 occurred only in the 2040 time step. As mentioned in Section 3.6, we selected six *Pucaras* from this list, i.e., Pi25, Pi20, Pi19, Pi16, Pi12 and Pi10, to conduct the UAV flights and derive the stream power index. This is presented in the next section.

**Table 6:** Most frequent modelling centers for *Pucaras* that headed the first three positions of maximum erosion.

Code	Future climate—modeling centers (count)				Total
	IPSL-CM6A-LR	CNRM-CM6-1	MIROC6	GFDL-ESM4	
**Pi19**	4	4	4	0	12
**Pi25**	4	4	4	0	12
**Pi12**	3	3	0	0	6
**Pi16**	1	1	4	0	6
**Pi10**	0	0	0	3	3
**Pi18**	0	0	0	3	3
**Pi20**	0	0	0	3	3
**Total**	12	9	12	12	

**Table 7:** Most frequent SSPs and time periods for *Pucaras* that headed the first three positions of maximum erosion.

Code	Future climate—modeling scenarios (count)							
	SSP				Time period			
	1–2.6	2–4.5	3–7.0	5–8.5	2040	2060	2080	2100
**Pi19**	3	3	3	3	0	0	2	10
**Pi25**	3	3	3	3	0	0	4	8
**Pi12**	2	1	1	2	0	0	2	4
**Pi16**	1	2	2	1	0	0	1	5
**Pi10**	1	0	1	1	3	0	0	0
**Pi18**	1	0	1	1	3	0	0	0
**Pi20**	1	0	1	1	3	0	0	0

### 4.4 Assessment of high spatial resolution DTMs and gully-prone areas in prioritized *Pucaras*

**Fig 7:**
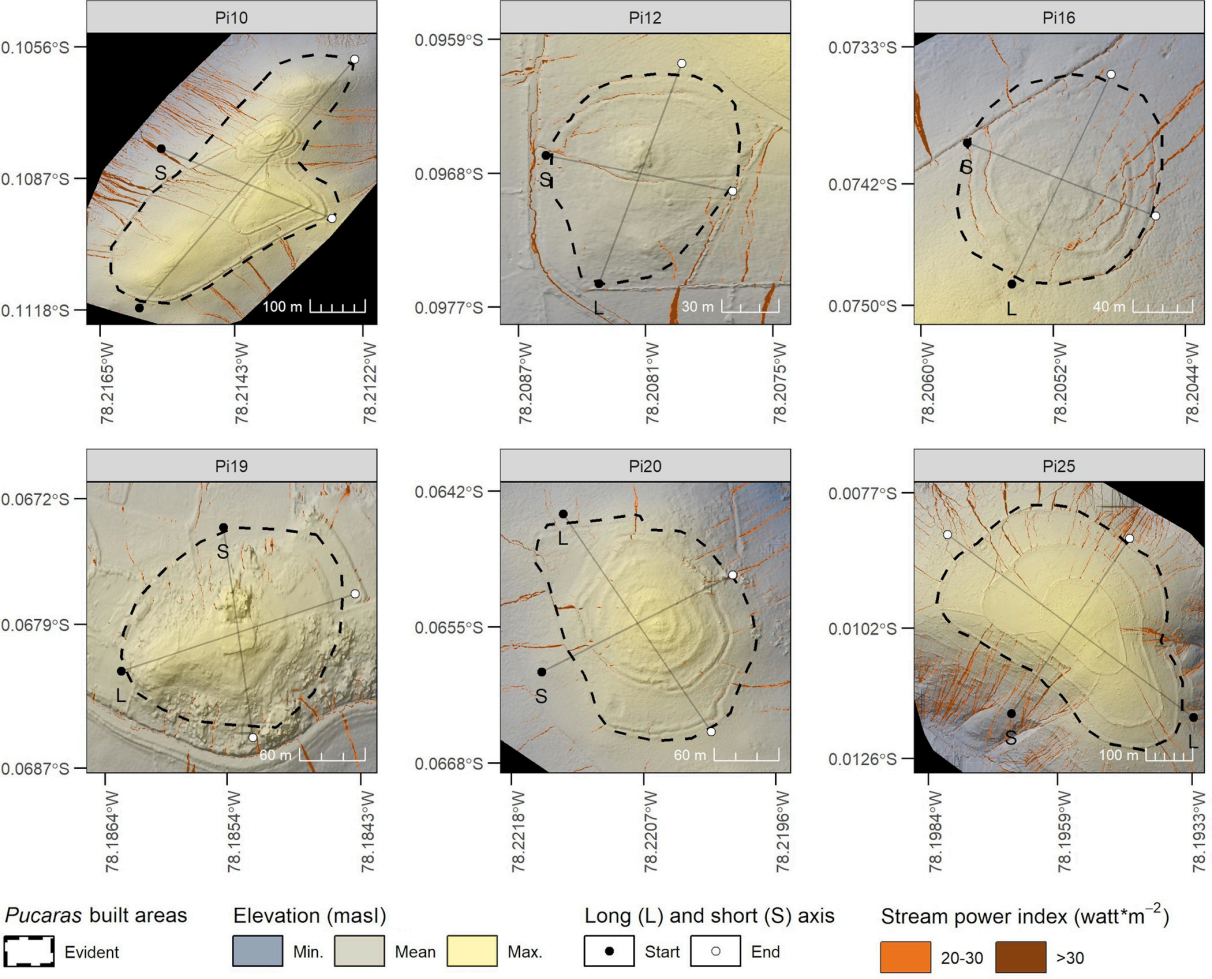
High spatial resolution DTMs, gully-erosion-prone areas and axes for the evident built area of *Pucaras*.

**Fig 8:**
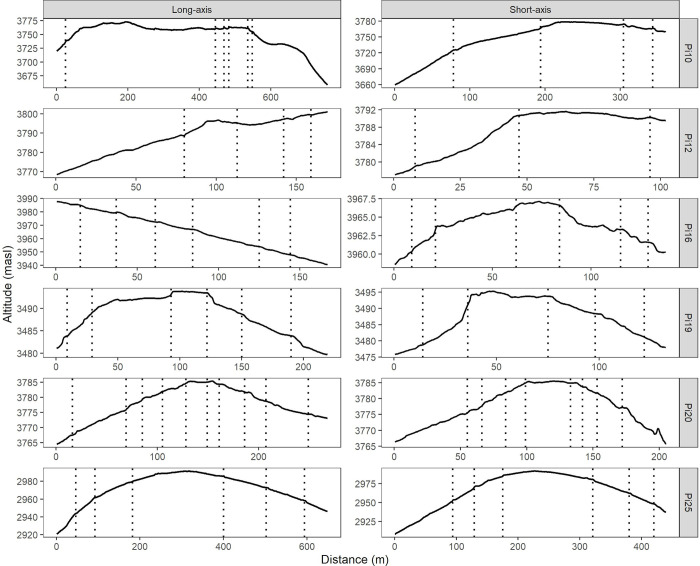
Elevation profiles derived for the ellipsoid axes of the prioritized *Pucaras*. Dotted lines indicate the position of terraces.

After prioritizing the *Pucaras* with the highest potential of increased soil erosion due to climate change, we now describe their gully-erosion-prone areas that were identified. This is shown in Fig 7, where the high spatial resolution DTMs and their altitudinal gradients (higher areas are shown in yellow, while lower areas are shown in blue) obtained from the prioritized *Pucaras* are plotted along with their erosion-dominated (>20 watt*m^-2^) areas. In these surfaces, some linear patterns are evident, especially in Pi12 and Pi16 since they correspond to irrigation canals, natural fences or roads. Moreover, some remaining artifacts such as trees do not seem to be completely removed in Pi16 (rough surfaces). Despite these limitations, gully-prone areas are still appreciable in all *Pucaras* as brown lines. In Pi10, Pi19, Pi20 and Pi25, they seem to follow a downward radial pattern, thickening each time they move away from the highest areas. This is different in Pi12 and Pi16, as they are on a slope and the gully-prone areas follow a linear pattern that also thickens in the lower parts. Of all the *Pucaras*, it can be observed that Pi10 has multiple gully-prone areas, especially on its northwest slope. The same occurs with Pi25, but more intensely and on its southwest, north and east slopes. Curiously, the concentric terraces of Pi20 and Pi16 seem to break the flow of some gully-prone areas. The locations of these terraces are better illustrated as dotted lines in the profiles shown in Fig 8, where the short and long axes of each *Pucara* are shown. Therefore, it is possible to corroborate that Pi10 has more gully-prone areas on its northwest slope, as its short axis seems to be long and steep. A similar situation is also observed for the long axis of Pi16 and Pi25, the latter also for its short axis. Despite the fact that, in most *Pucara* cases, the terraces are not totally appreciable in these profiles, some of them are. In Pi20, for example, at least ten were counted for its long axis, while for its short axis, eight were counted. Moreover, the distances among them seem to be the shortest among all *Pucaras*, which could be related to the broken flow of the gully-prone areas previously shown.

To better account for this, Table 8 summarizes the gully-prone areas in the evident built area of each *Pucara*, together with the number of terraces observed along their axes and the average distances between them. Therefore, it is possible to observe that Pi16 accumulates the largest area prone to gullies (3.28%), followed by Pi25 (2.89%) and Pi10 (1.93%). Even though Pi25 and Pi10 seem to have the largest distance between terraces, averaging 87 m (*SD* = 57) and 98 m (*SD* = 136), as well as a lower number of them considering their size (10 and 12 are counted in Pi25 and Pi10 along their axis distances ranging from 360 to 761 m), Pi16 seems to have shorter distances between terraces accounting an average of 24 m (*SD* = 136), but they are more numerous considering its size (also 12 terraces but spread over a shorter axis distance ranging from 137 to 167 m). Due to its characteristics, Pi16 seems more similar to Pi20, Pi19 and Pi12, whose terrace distances range from 21 to 33 m and account for between 7 and 20 terraces over axis distances ranging from 101 to 268 m; nevertheless, it has a lower gully-prone area proportion (<1%). This higher vulnerability of Pi16 to gullies should be put in context as it is located on a steep slope (as shown in Fig 8); therefore, it is different to these *Pucaras* as they are mostly located on moderate slopes (Pi12) and in relatively flat areas (Pi19 and Pi20).

**Table 8:** Summary of erosion-prone areas identified with stream power index, together with terraces number and their ellipsoid axes distances for the prioritized *Pucaras*.

Code	Stream power index (watt*m^-2^) areas^a^						Terraces			Axes distances	
	20–30		>30		Total		Number	Average distance among them (m)		Long	Short
	(m^2^)	(%)	(m^2^)	(%)	(m^2^)	(%)	(count)	Mean	SD	(m)	(m)
**Pi16**	214.4	1.27	338.1	2.01	552.5	3.28	12	24	9	167	137
**Pi25**	2164.1	1.30	2626.8	1.59	4790.9	2.89	12	87	57	649	440
**Pi10**	1208.0	0.84	1555.9	1.09	2763.9	1.93	10	98	136	761	360
**Pi20**	173.0	0.49	203.6	0.58	376.7	1.08	18	21	11	268	204
**Pi19**	100.2	0.51	87.2	0.45	187.5	0.97	11	32	13	218	132
**Pi12**	62.2	0.52	48.1	0.41	110.3	0.94	7	33	11	170	101

^a^ Percentage calculated from the evident built area of *Pucaras*.

## 5.Discussion

### 5.1 Strengths and limitations of modeling rain erosion with RUSLE

We applied the RUSLE at the regional scale, using various data sources for a historical climate model but also for multiple future climate models. This allowed us to calculate rain erosion for a base period but also to obtain an approximation of future impacts on built heritage in the Pambamarca study area. In this sense, we found that *Pucaras* Pi25 and Pi19, which are located on the eastern and northeastern slopes of Pambamarca, report a medium erosion rate for the historical climate model (10–20 ton*ha^-1^*yr^-1^) and a higher probability of increasing their erosion rates in the coming decades (ranging between 0.3 and 4 ton*ha^-1^*yr^-1^) according to the future climate models that reported more rainfall (i.e., IPSL-CM6A-LR, MIROC6 and CNRM-CM6-1). The rest of the Pucaras which are located on the central and western slopes reported low erosion rates (<10 ton*ha^-1^*yr^-1^) but also increased erosion rates (ranging between 0.1 and 3 ton*ha^-1^*yr^-1^) according to the future climate models. These erosion rates at *Pucara* sites are more conservative than those described by Ochoa-Cueva et al. [110] for vegetated areas in the south Andes of Ecuador but on steeper slopes and for higher rainfall intensities (15 to 40 ton*ha^-1^*yr^-1^). More closely resembling our erosion rate estimation is that reported by Henry et al. [111] in central Ecuador, which varied around 10 ton*ha^-1^*yr^-1^ in the *Páramo* grasslands (recall that the *Pucaras* are mostly located in this ecosystem), increasing from 50 to 150 ton*ha^-1^*yr^-1^ for pasture and annual cropping areas, respectively. Although future climate models were not evaluated in these studies, other research applying them (e.g., HadGEM2-ES, HadCM3) reported mixed impacts on the RUSLE, with soil loss ranging between -25% and 25% of the baseline scenarios [112,113]. This is comparable to our results, as, depending on the climate model applied, soil loss magnitudes tended to increase or decrease depending of the *Pucara* evaluated (e.g., Pi25 varied between -13.9% to 34.8% considering the rainiest and driest climate models). Although our results seem to agree with the literature and allow us to identify *Pucara* sites at risk of rain erosion, it is equally important to mention that the qualitative assessment reported a moderate score of 0.62 for the Kappa index. The achievement of this score involved different reasons, the first being the difficulty in qualitatively differentiating the rate of erosion present at a site despite our approach using multiple geophotos acquisition techniques. With respect to the quantitative assessment, we found that only one of the three correlation tests was statistically significant. In this case, the limited number of soil samples due to difficulties in collecting them in the field (e.g., samples on private properties, distant location of sampling points, local soil variation) did not allow us to corroborate the negative correlation we expected with fine textures. Other authors reported advantages using high-resolution satellite images to identify gullies, especially for those related to tillage practices [114]. However, remote sensing techniques are limited to observing the land cover from above, and thus it can be difficult to identify subtle erosion evidence such as pedestaling of plant crowns or small rills. Thus, combining different validation techniques is a recommended practice that is also well perceived in the literature [115] and implemented in this research. A second point to take into account in our results is related to the downscaling procedure. Although the application of machine learning algorithms such as Random Forest [116,117] is a recognized and common procedure to improve the spatial resolution of soil [118] or rainfall [119] features, it is not guaranteed that local variability is represented correctly. This conflicts with data acquired at the field scale and used for validation. A third factor is the limitation of RUSLE as a methodology. The RUSLE is an equation that estimates average annual soil loss by sheet and rill erosion on those portions of landscape profiles where erosion, but not deposition, occurs. It does not estimate deposition like that at the toe of concave slopes, and it does not estimate sediment yield at a downstream location. Also, it does not include ephemeral gully erosion. An important scientific limitation of the RUSLE as an empirically based equation is that it does not represent fundamental hydrologic and erosion processes explicitly. For example, the effect of runoff, as might be reflected in a hydrologic model, is not represented directly in the RUSLE. Fundamental erosion processes and their interactions are not represented explicitly. An example where the RUSLE does not give the proper result is the deposition of sediment in furrows on flat grades. Analysis of any single data set may show significant differences between estimates with the RUSLE and observed data. Such limited comparisons are not necessarily an indication of the overall performance of the RUSLE. As an empirical equation derived from experimental data, the RUSLE adequately represents the first-order effects of the factors that affect sheet and rill erosion [11,12]. This may explain why it was difficult to discern between the medium and high erosion categories in the field. While our objective was to identify erosion risks rather than sediment dynamics, new methodologies such as Unit Stream Power Erosion and Deposition (USPED) [120] and many others [121] offers methodologies for fill this gap. Future work and applications are also encouraged to investigate wind erosion, which is a factor that has not been taken into account in this study but is certainly important in the study area.

### 5.2 Future climate models’ variability and assessment of their impacts in soil loss prediction

We experimented with different future climate models to model soil loss with the RUSLE. This provided us with unexpected outputs which allowed us to dimension probable impacts in terms of soil loss rates. As mentioned above, Pi25 and Pi16 stood out for their medium erosion rates with the historical climatic model, but when we look at the future climate models, they could change their erosion category from medium to high, similar to other *Pucaras* with significant erosion rates (e.g., Pi12 and Pi16). This ranking depended more on the selection of the modeling institution than on the selection of the SSP, but the *Pucaras* mentioned above turned out to be the most affected by their frequent occurrence in the different future climate models. With regard to these models, models such as IPSL-CM6A-LR, CNRM-CM6-1 and MIROC6 showed an increase in rainfall predictions, with the first one being the most extreme (39.1% more rainfall in our study area for SSP5-8.5, time step 2080–2100). On the other side, GFDL-ESM4 projects a reduction in precipitation (-15.7% also for SSP5-8.5, time step 2080–2100), leading to a decrease of soil loss. This high variability among models, even considering the similar simulation parameters (e.g., SSPs, time steps), added an important layer of uncertainty that could not be easily understood until the model was applied and compared. A similar finding was also reached by Vetter et al. [122], who considered that the selection of a global climate model from CMIP5 causes the greatest uncertainty compared to its emission scenarios (RCP), especially at local scales. Although the new generation of CMIP6 models improve the narrative of their emission scenarios (or SSPs), it is important for researchers to select more than one model and SSP after a literature review. It is then recommended to have ensembles of runs to decide which model (or models) could better describe future climate conditions. Similar recommendations are also found in the literature [123,124], but new procedures to select models after testing should also be advised [125–127]. With respect to SSPs, it is important to mention that their impact on soil erosion was constant in some cases but more variable in others. The latter was the case of MIROC6, whose impact on soil erosion appeared positive or negative depending on the SSP and the selected time step. Other models, regardless of the SSP selected, showed an impact in one direction, being positive in IPSL-CM6A-L and CNRM-CM6, and negative in GFDL-ESM4. Although ensemble means are mentioned in literature to cancel systematic errors of individual models [128,129], in our case, it was more interesting to identify which models suggested the extreme climate scenarios. This provided us with more elements to judge the effects of medium- and long-term rain erosion, which also guided our prioritization procedure based on ranking rates and measuring models’ occurrences. This was better than relying on assessments of general circulation models which can be subjective and vary locally [130], and concentrating more on observing the effects of climate extremes.

### 5.3 Identified gully-prone areas and opportunities using UAV in heritage preservation

We prioritized *Pucaras* at risk of soil loss due to climate change impacts and conducted UAV flights to derive high-resolution DTMs of the most affected. Our flight campaigns allowed us to zone areas with a risk of gully formation in six of the seven prioritized *Pucaras*. We found that Pi16, Pi25 and Pi10 presented the highest percentage of area at risk of gullying, with this ranging from 1.9 to 3.8%. Of these, Pi25, followed by Pi10 and Pi16, presented the largest area at risk, with 4790.9, 2763.9 and 552.5 m^2^, respectively. Although some artifacts remained in the DTMs (e.g. irrigation canals, natural fences, roads) that may have affected the identification of the gullies, the area found is less than 15% of the total area analyzed, a figure that is rarely exceeded according to the literature [131]. In comparison with other DTMs of medium-resolution, we observed that for Pi25 (See S1 Fig), the SRTM-derived stream power index averaged 0.04 (*SD* = 0.03), the ASTER-derived [132] one averaged 0.007 (*SD* = 0.005), while the UAV-derived one averaged 3.6 (*SD* = 13.8). These figures demonstrate that the gullies were practically invisible to medium-resolution DTMs at the scale of *Pucaras* analysis; however, acquiring these high-resolution DTMs are certainly demanding in terms of field work, requiring in our case at least six days of optimal flight conditions (See Section 3.6), but also computer processing power (e.g. Pi25 took 45 min for processing in a Intel core i7-8700 CPU with 40 GB RAM) to analyze them. Nevertheless, the field visit to each of the *Pucaras* flown over allowed us to identify other specific factors that reflected our results. The linear features observed in Pi12, Pi16 and Pi20, for example, indicated that furrow irrigation, short-distance terraces and natural barriers conducted water flow, relocating or even decreasing the risk of gullies. This was also concluded by Vanacker et al. [133] for a study area in southern Ecuador, but for a basin with semi-arid to arid climatic conditions. The latter, together with geomorphological factors, were discussed as more important drivers for land degradation than those derived from grazing in similar regions of the Andes [134]. This statement is also relevant in our case, as we observed that Pi25 reached the highest gully risk by being located at the lowest elevation, where soils are shallow, easily erodible and mostly degraded (See Section 2.2). On the contrary, Pi10, whose surrounding area is better conserved and declared for communal conservation by the Socio Bosque Program [135], also presented a high risk to gullies due to its sloping areas. However, since our approach uniquely relied on high spatial resolution DTMs to better observe the geomorphological susceptibility to gully erosion, their conjunction with climate, edaphology and land cover is mandatory to adequately address any land degradation process. A combination of UAV-derived products (e.g., DTMs, multispectral photography) with regional-scale models (e.g., RUSLE, future climate) is what we recommend as an opportunity in the preservation of built heritage. To be more specific, high spatial resolution DTMs and the derivation of gully-prone areas in those prioritized *Pucaras* reduced our need for more flight campaigns and field visits but also complemented and refined our observations regarding soil loss and climate change impacts. The potential of these synergies is still under-exploited [136], but users interested in this methodology should be aware of some recommendations with respect to the selection of the UAV. As *Pucaras* and similar sites are usually remotely located, a lightweight drone with an easy calibration and ideally waterproofed can make a difference. However, in mountainous regions, the power of the drone must be taken into account, as the wind at the summits is often intense (in Pi14, we experienced gusts of more than 10 m s^-1^); therefore, flight planning is needed to match less windy and wet seasons. In this context, drone design workflows to inventory and monitor specific indicators are needed [137]. Such workflows should also consider techniques for emergency landing in adverse situations (e.g., sudden fog or rain, wildlife stress, lose line of sight) to avoid accidents or injuries [138,139], but they should also consider other alternatives when surveying with a UAV is not feasible [140].

### 5.4 Recommendations for *Pucaras* management in the context of climate change

Throughout this work, we analyzed the soil loss risk of *Pucaras* of the Pambamarca region and identified those that should be managed in the context of climate change. At the local scale, we also identified the areas prone to gullying and observed the topographic characteristics of the *Pucaras* that help to reduce erosion. In this sense, it is important to highlight that the level of risk depends very much on the conjunction of the factors previously mentioned, i.e., climate, soils, topography and human impact, which in fact constitute the dimensions included in the RUSLE. However, as soil erosion takes place when catastrophic and moderate rainstorms occur, especially in semi-arid areas [141,142], and climate change variability is linked to an increased probability of such events [143], a soil conservation strategy seeking the preservation of built heritage should consider these dimensions. Therefore, observing in Table 9 we summarize our proposal based on rain erosion susceptibility for each *Pucara* in-situ and context areas. These strategies are divided into measures which are summarized from Morgan [144] and Carrasco-Torrontegui [145], the latter study being related to ancestral practices of Andean indigenous populations. On the other hand, considering that the Cultural Heritage Law of Ecuador mentions in its seventh article that archaeological monuments are part of the State’s Cultural Heritage [90], we also present some guidelines related to this law regarding the in-situ preservation of the *Pucara* sites.

**Table 9:** Suggested strategies based on susceptibility to rain erosion.

Ecosystem, altitude range (masl)	Code	Estimate managed area (%)^a^	Intervened area (%)^b^	Sheet and rill erosion^c^	Gully formation^d^	Climate change variability^e^	Management strategies
***Páramo* grasslands** **(4078–3720)**	Pi13	0	0	++	-	+	P, S
	Pi14	0	0	+	-	+	P
	Pi16	0	0	++	+++	++	P, S
	Pi15	SB-I (34%)	0	++	-	+	P, S
	Pi17	0	0	++	-	+	P, S
	Pi10	SB-C (98.2%)	4.8	+	++	++	P, A, S
	Pi12	0	84.2	++	+	++	P, A, S
	Pi20	0	33.8	+	++	++	P, A, S
	Pi18	SB-I (100%)	0	+	-	++	P, S
	Pi11	0	100	+	-	+	P, A
**High Montane Evergreen Forest** **(3614–3400)**	Pi23	0	88.6	++	-	+	P, A, S
	Pi21	SB-I (7.7%)	92	++	-	+	P, A, S
	Pi19	0	100	+++	+	+++	P, A, S
	Pi22	0	100	++	-	+	P, A, S
**Northern Semi-deciduous Forest and Shrubland of the Valleys (<2997)**	Pi25	0	0	+++	+++	+++	P, A, S, M

^a^ Obtained from protection categories by MAE [146]. Acronyms used stand for: SB-C, Socio Bosque community land; SB-I, Socio Bosque individual land; and N, without a protection category

^b^ Derived for the *Pucara* buffer areas using a 2018 land cover map [147] and merging the classes: Croplands, populated area, and infrastructure

^c^ Based on the historical mean annual soil loss ranges (10–20:+++; 5–10:++; 1–5:+)

^d^ Based on the stream power index area percentages (>2%:+++; 1–2%:++; <1%:+)

^e^ Based on the top three position in models count (>7:+++; 3–6:++; <3:+)

^f^This strategy was included even though its declared intervened area is 0, since our field observations suggest agricultural activities in the context area of *Pucaras*.

Four groups are listed:

- Agronomic measures (A), which utilize the role of vegetation to protect the soil against erosion in the context area of the *Pucara*. These are less expensive and directly deal with reducing the raindrop impact, increasing infiltration, reducing the runoff volume and decreasing wind and water velocities. Some examples are crop management (high-density planting, multiple cropping, cover cropping), agroforestry, vegetation restoration (plant trees, shrubs, grasses), mulching, biological engineering and hydroseeding.
- Soil management (S), which is concerned with ways of preparing the soil to promote plant growth and improve its structure so that it is more resistant to erosion. It targets the context area of the *Pucara* and some examples are organic content enrichment, conservation tillage (contour, ridging, minimum), drainage management and soil stabilizers.
- Mechanical measures (M), which involve engineering structures and depend on the manipulation of the surface topography in the context area of the *Pucara*. Its main function is to complement agronomic and soil measures when they are not sufficient, being used to control the flow of any excess water and wind that may arise. Some examples are contour bunds, terraces, raised beds (*waru-waru*), waterways (*qochas*), stabilization structures (sidewalls, embankments, cuttings, footpaths), geotextiles, bush matting and windbreaks.
- Preservation measures (P), which involve activities or initiatives to preserve the in-situ area of the *Pucara*, which should be carried out in coordination with the Institute of Cultural Heritage of Ecuador. Some examples are research (involving archeological and paleontological excavations), restoration, exhibition and promotion of built heritage areas, enact regulations, denunciation of prohibited activities (e.g. trafficking of archeological pieces, looting, mining, etc.), impose precautionary measures or even expropriate the State’s Cultural Heritage if it is at eminent risk of destruction.

Although no climate change adaptation strategy is mentioned here, it should be mentioned thatthe abovementioned measures contribute to reducing the impacts of soil loss due to climate change. However, as the Pambamarca region is characterized by agriculture, and climate change will exert more pressure to expand the agricultural frontier [148], it should be noted that the occurrence of events such as fires, overgrazing and total/partial destruction of the *Pucaras* is likely. In this sense, preservation measures symbolized the first protection layer for preserve intact as much as posible, all *Pucaras* sites with no exception. These measures should not exclude the communities that maintain their culture and history around the Pamabamarca Fortress Complex, but rather recognize the fragility of these landscapes and therefore promote measures that strengthen the preservation of the heritage without ignoring the development to which their communities also aspire. In this sense, the Biocultural Heritage [149] is an interesting approach, as it brings together archaeological, architectural and heritage heritage rather than in isolation. In this sense, land use practices are important indicators of adaptation to climatic extremes, so the study of the role of these *Pucaras* terraces in reducing erosion is highly recommended [150]. Further south in Ecuador, e.g., the *Inka Pirka*, "Inca wall" constitute a complex of castles of Cañari-Inca origin, whose terraces combine agricultural land, ethnobotanical plantations, edible gardens and other plants uses [151]. These practices and knowledge are invaluable assets for future generations whose preservation should be guaranteed. Our assesment of the management of the *Pucaras* reveals that only Pi10 and Pi18 are covered by the Payment of Enriomental Services (i.e. Socio Bosque program) [146]. This lag in the management of the Pambamarca Fortress Complex is of concern, because promoting other initiatives for the preservation of Pucaras requires financing structures where the local government promotes subsidizing the conservation of the Pambamarca Fortress Complex. These initiatives should guarantee to peasant families and other local stakeholders the custody of the archaeological site, with the objective of generating income from tourism and the provision of local food, water and refreshments to visitors [152]. In addition, these strategies should include the effects of climate change in the Pambamarca region, especially for the fragile *Páramos* grassland ecosystem (See section 2.2), and its degradation implications for water supply [153]. Although these coping strategies are beyond the scope of this research (interested readers can refer to [154,155] to review more strategies), the government of Ecuador has outlined strategic guidelines and financing mechanisms for the agricultural and water sectors, as well as for other priority sectors in relation to adaptation and mitigation of climate change impacts [156,157]. Finally, the strategies mentioned in Table 9 were formulated based on the risks and the intensity of erosion degradation currently observed; however, the preserve measurements should be discussed and analyzed on a case-by-case basis, with national and local authorities. Therefore, the information generated in this research (See S2 Fig, S2 and S3 Files) could be useful to promote preservation of more *Pucaras*, but it will also help to discuss management strategies.

## 6. Conclusion

This study presented the challenge of preserving built heritage due to rain erosion and climatic impacts in the Pambamarca Fortress Complex and its *Pucaras*. We developed a methodology that combines the RUSLE, climate models and UAV imagery to prioritize and identify which *Pucaras* are and will be prone to sheet, rill and gully erosion. To this effect, we demonstrated how rain erosion could be intensified or diminished by climate change in *Pucara* areas in order to carefully observe which of them should be monitored and managed. The results of this assessment helped us to conduct a series of flight campaigns to observe, in detail, the topography of the *Pucaras* at risk and identify their gully-prone areas from high-resolution DTMs and the stream power index. A surprising and unprecedented result of this analysis was the effect of the *Pucara* terraces in deterring gully erosion, which suggested the implementation of strategies to protect soils from erosion by their architects and builders. This led us to suggest some strategies and measures to protect them in a specific way that include mechanical, agronomic, soil and preservation management measures. The importance of these strategies is to find and discuss mechanisms for adaptation to climate change but also to highlight conservation gaps to preserve this built heritage and its landscape.

## Supporting information

S1 Fig Stream power index derived from SRTM, ASTER and UAV for the Pi25 *Pucara*. (JPG)

S2 Fig Minimum spatial data sets in raster and vector formats to run S3. (TIF)

S1 Data (ZIP)

S1 File (R)

S2 File Routine to reproduce figures and graphs. (ZIP)

S3 File Routines for RUSLE calculation. Usage and data sets not included in S2 to run these routines are available from the authors upon request.
(JPG)
