# Supplementary figures and images for: Impacts of soil erosion and climate change on the built heritage of the Pambamarca Fortress Complex in northern Ecuador
Source: PLoS One. 2023 Feb 23;18(2):e0281869. doi: 10.1371/journal.pone.0281869 (PMC9949680; doi:10.1371/journal.pone.0281869)

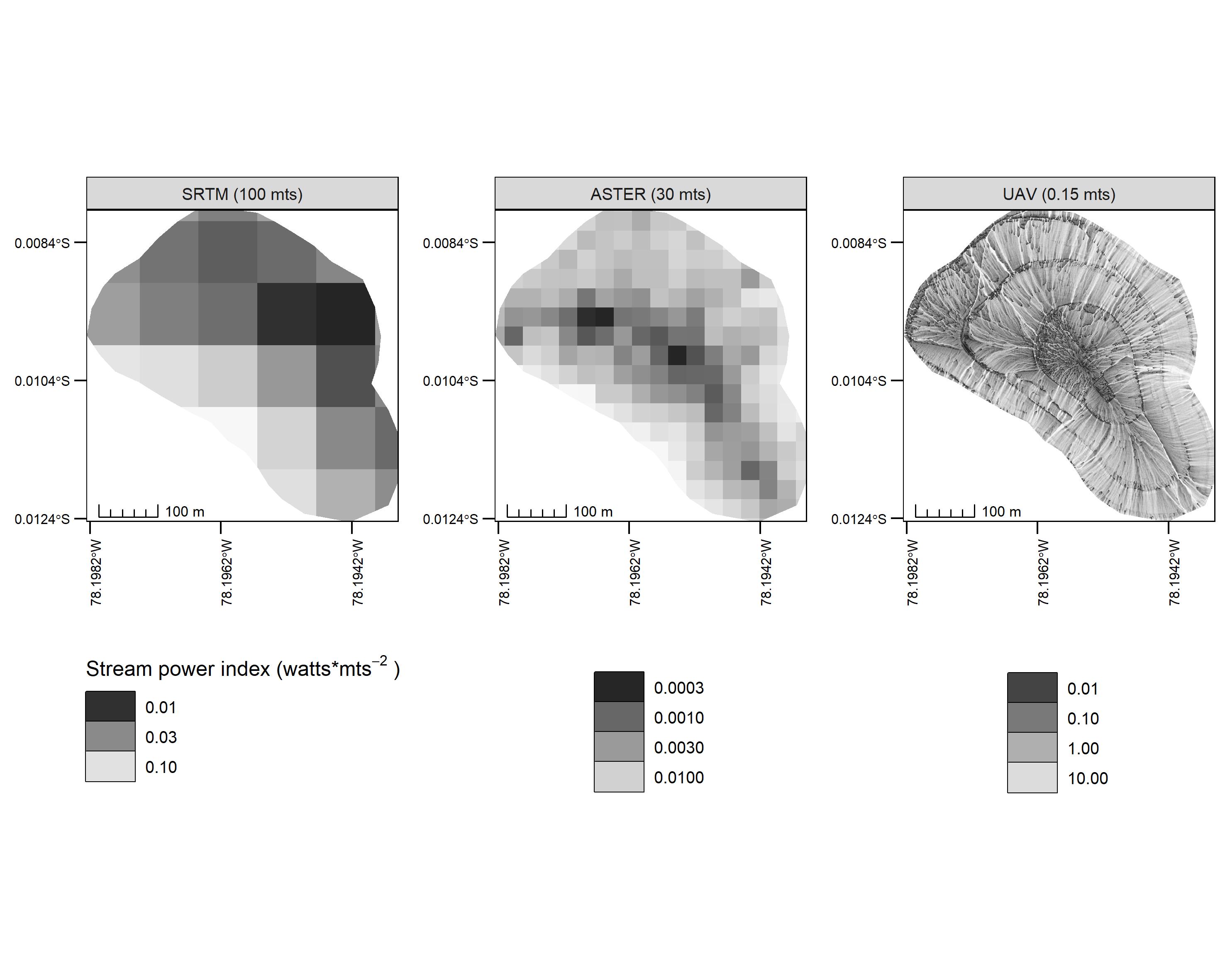

Supplement: S1 Fig — (JPG) [file pone.0281869.s001.jpg]

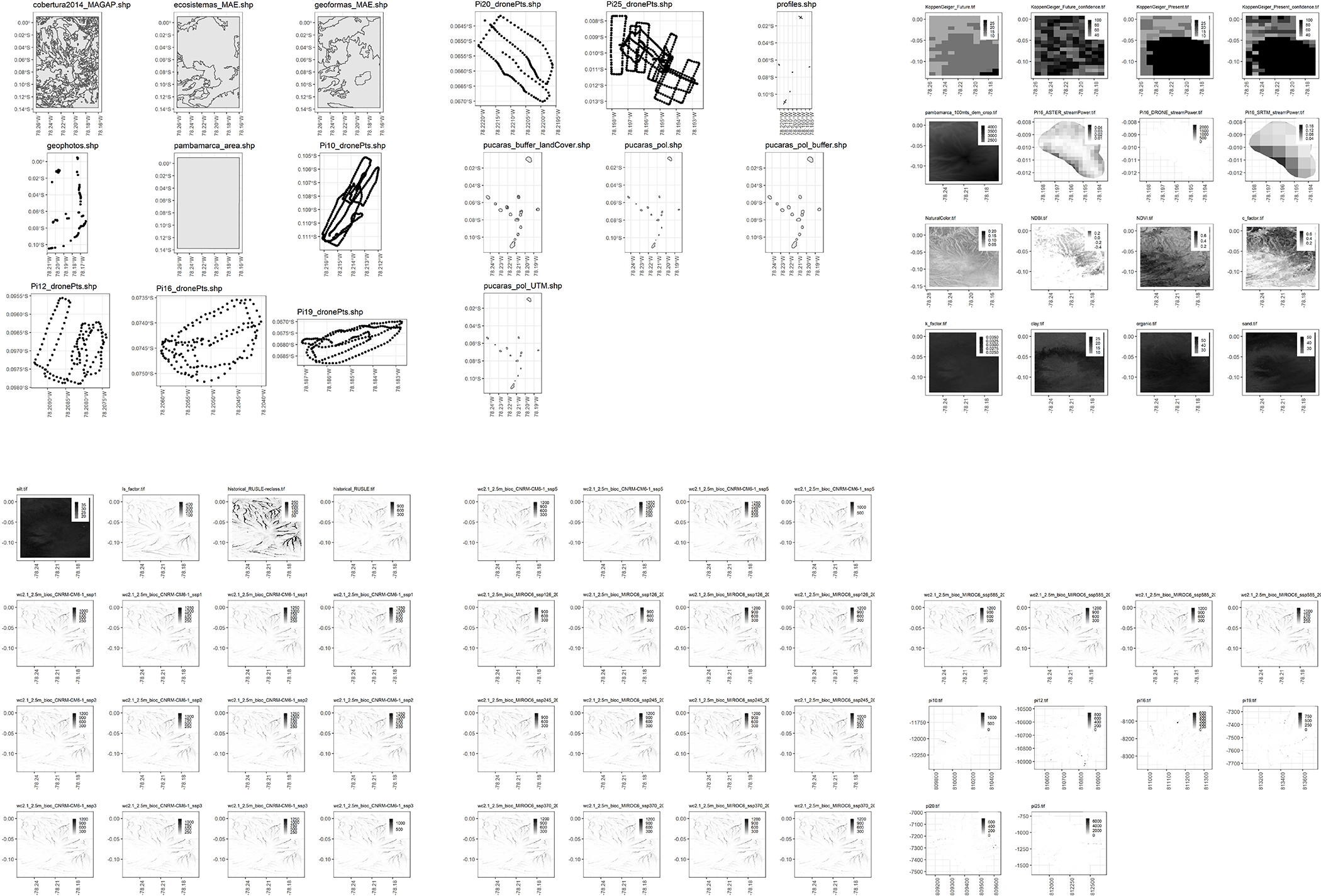

Supplement: S2 Fig — (TIF) [file pone.0281869.s002.tif]

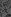

Supplement: S1 Data — (ZIP) [file pone.0281869.s003.zip › data/characterization/KoppenGeiger_Future_confidence.tif]

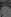

Supplement: S1 Data — (ZIP) [file pone.0281869.s003.zip › data/characterization/KoppenGeiger_Present_confidence.tif]

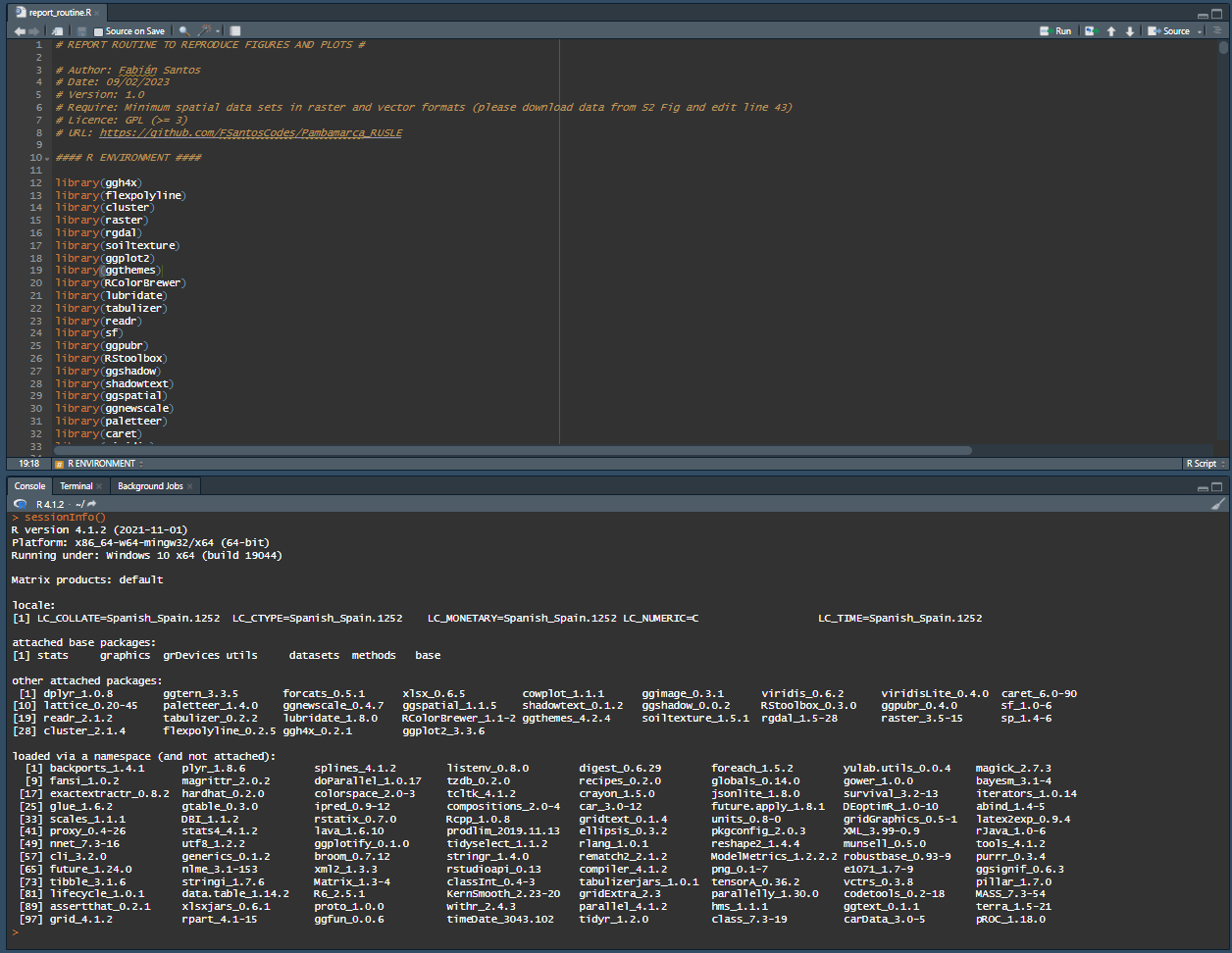

Supplement: S3 File — Usage and data sets not included in S2 to run these routines are available from the authors upon request. (JPG) [file pone.0281869.s006.jpg]
