# Supplementary material for: Impacts of soil erosion and climate change on the built heritage of the Pambamarca Fortress Complex in northern Ecuador
Source: PLoS One. 2023 Feb 23;18(2):e0281869. doi: 10.1371/journal.pone.0281869 (PMC9949680; doi:10.1371/journal.pone.0281869)
Supplement: S1 Data — (ZIP) [file pone.0281869.s003.zip › data/references/Connel_Pambamarca.pdf]

Inka Militarism at the Pambamarca Complex in Northern Ecuador

Samuel V. Connell, Amber Anderson, Chad Gifford, and Ana Lucía González

In this article, we present research on Inka actions in the face of resistance by indigenous peoples on the northern frontier. We link fieldwork at the Pambamarca complex in northern Ecuador with historic documents to provide important context for further examining imperial processes. With its three site types, Pambamarca offers an opportunity to examine the range of tendencies that groups undergo during imperial moments. Its sites show evidence of both direct displays and the materialization of forceful control or takeover, as well as the more passive, nonsettler, decentralized hegemonic narratives also commonly associated with empire. Here we present detailed data for Inka military installations used to confront a prolonged resistance by the País Caranqui, a decentralized confederation of Caranqui-Cayambe peoples. Evidence from surveys and excavations—including architectural planning, distribution of artifacts, and military encounters—at two large sites in the complex, Quitoloma and Campana Pucara, helps expand our current understandings of the Inka invasion in northern Ecuador while broadening our perspective on the imperial narrative in South America.

Keywords: Pambamarca, Inka, Ecuador, militarism, frontier, pucara, Cayambe, País Caranqui

En este trabajo se presentan las investigaciones realizadas sobre la resistencia indígena contra el militarismo incaico en la Sierra Norte de Ecuador. Se vincula el trabajo de campo realizado en el complejo de Pambamarca con documentos históricos que proporcionan un contexto importante para comprender con más detalle el proceso imperial. A través de sus tres tipos de sitios, Pambamarca ofrece una oportunidad para examinar la gama de tendencias que desarrollan los grupos durante momentos imperiales. Los sitios en Pambamarca muestran evidencia tanto de manifestaciones directas y de la materialización del control forzoso como de narrativas hegemónicas más pasivas sobre la descentralización no colonial que también son comúnmente asociadas con un imperio. Aquí presentamos datos detallados de las instalaciones militares incaicas construidas para enfrentar una resistencia prolongada por la confederación descentralizada de grupos Caranqui-Cayambe, llamados colectivamente País Caranqui. La evidencia procedente de sondeos y excavaciones que se realizaron en dos de las fortalezas del complejo Pambamarca, Quitoloma y Campana Pucará, incluyendo la planeación arquitectónica, la distribución de artefactos y encuentros militares, ha contribuido a ampliar nuestro conocimiento sobre la invasión incaica en el norte del Ecuador y a incrementar nuestra perspectiva sobre la narrativa imperial en Sudamérica.

Palabras clave: Pambamarca, Inca, Ecuador, militarismo, frontera, pucara, Cayambe, País Caranqui

In his recent call for a multidisciplinary approach, Shimada (2015:2) emphasizes a holistic and integrated vision of the Inka Empire that views provincial areas with similar interest as the heartland. At the turn of the sixteenth century on the northern frontier of Ecuador, the Inka emperors sought to extend their influence and establish a new base of power (Bray 2015). They built a series of fortifications in the late 1400s to combat a lengthy resistance by indigenous Caranqui-Cayambe groups forming the País Caranqui, which thwarted Inka movement northward (Athens 1978, 1992; Betanzos 1987[1551–1557]; Bray 1991, 1992,

Samuel V. Connell ■ Department of Anthropology, Foothill College, 12345 El Monte Road, Los Altos Hills, CA 94022, USA (connellsamuel@foothill.edu, corresponding author)
Amber Anderson ■ Department of Sociology and Anthropology, Rochester Institute of Technology, 92 Lomb Memorial Drive, Eastman Hall, Suite 1301, Rochester, NY 14623, USA
Chad Gifford ■ Columbia University, 403 Lerner Hall, MC 1201, 2920 Broadway, New York, NY 10027, USA
Ana Lucía González ■ Department of Anthropology, Foothill College, 12345 El Monte Road, Los Altos Hills, CA 94022, USA

Latin American Antiquity 30(1), 2019, pp. 177–197
Copyright © 2019 by the Society for American Archaeology
doi:10.1017/laq.2018.80

Downloaded from https://www.cambridge.org/core. University of New England, on 28 Mar 2019 at 21:59:08, subject to the Cambridge Core terms of use, available at https://www.cambridge.org/core/terms. https://doi.org/10.1017/laq.2018.80

2008; Cabello Valboa 1951[1586]; Caillavet 2000; Cieza de León 1959[1553]; Cobo 1979 [1653]; Cordero Ramos 1998; D'Altroy 1992, 2002; de la Vega 1961; Espinosa Soriano 1988; Fresco et al. 1990; Hyslop 1990; Jijón y Caamaño 1952; Montesinos 1957[1644], 2010; Oberem et al. 1969; Ogburn et al. 2009; Plaza Schuller 1976, 1977, 1978; Ramón Valarezo 1987; Salomon 1986; Sarmiento de Gamboa 2007[1572]). As Father Bernabé Cobo related,

> The Indians of those provinces were brave and warlike, and many times the troops of the Inca were defeated and routed, and not a few times the king himself fled. Cayambes, particularly, being men of valor and courage, made it difficult for the Inca Guayna Capac and his captains that in conquering them a great deal of time and blood were lost [1979(1653):157].

Situated just south of Cayambe, the Pambamarca project area reveals this story of resistance and provides strong historical context to examine imperial formations—what Lori Khatchadourian (2016) aptly calls the "process of becoming" in her study of satrapy in the Persian Empire (see Stoler 2008; Williams 1978)—and to move beyond "testing ethnohistorical assertions to address aspects of Inka imperialism not recorded by early writers" (Shimada 2015:7). Work in Pambamarca is helping us understand how imperial forces and local groups reacted to and experienced tremendous change during prolonged conquest. Our expectation is that these imperial moments include militaristic displays of power and attempts at direct control incorporated with more indirect tendencies under conditions of partial sovereignty (Khatchadourian 2016), defined by "oblique relations to formal institutions" (Williams 1978:117; see Stoler 2008), through which the materialization of empire is negotiated at the level of indigenous communities. Native populations often shaped frontier dynamics because Inka control strategies were frequently in flux based on their desired wants or needs from an area (Alconini 2016). In Pambamarca we find evidence of different types of imperial debris: (1) constructions of three types of large impressive enclosures and roadways consistently associated with (2) direct evidence of military preparedness and conflict and (3) local settlement continuity with an Inka and Spanish overlay.

Given this palimpsest of overlapping data that cannot all be described here, this article focuses on the direct phase of imperial contact, invasion, and forceful resistance by describing archaeological investigations at two of the largest installations in Pambamarca. Evidence from these sites shows intensive Inka military activity, reinforced by physically imposing installations. Our work gives supporting evidence for the Inka incursion, struggles, and eventual victory over groups in northern Ecuador, but only provides the merest taste of the overall imperial narrative.

Background

It is not well defined when the Inka began to establish control in Ecuador, so our investigations employ Athens' (1978, 2003) chronology of a Late Period (AD 1250–1505/1525) followed by the Inka Period (AD 1505/1525–1532). During the Late Period, the northern Ecuadorian highlands contained a loose confederation of peoples sharing a common language and unity of cultural forms, known academically as the País Caranqui (Figure 1; see Athens 1992; Bray 1992, 2008, 2015; Ogburn et al. 2009; Salomon 1986). Tribes or chiefdoms (variably called *señoríos, cacicazgos, curacazgos, allyus*, or *parcialidades*), which from north to south included the Imbabura, the Caranqui, the Otavalo, the Cochasquí, the Cayambe, and the Guayllabamba, competed for power during this period (Caillavet 2000; Espinosa Soriano 1988).

The Inka are believed to have reached and conquered Quito in AD 1455–1460 under the reign of Topa Inka Yupanki. Huayna Capac led the Inka efforts to incorporate the peoples north of Quito during his reign from around AD 1490 until AD 1505/1525. Chroniclers allude to the importance of the region (Betanzos 1987 [1551–1557]:214–217; Cieza de León 1959 [1553]:261; de la Vega 1961:279; Espinosa Soriano 1988; Montesinos 1957[1644]:111; Murra 1947). The new territory would have provided many economic gains to the empire: northern Ecuador had fertile farm land and resources, particularly in the upper reaches of the rainforest,

Downloaded from https://www.cambridge.org/core. University of New England, on 28 Mar 2019 at 21:59:08, subject to the Cambridge Core terms of use, available at https://www.cambridge.org/core/terms. https://doi.org/10.1017/laq.2018.80

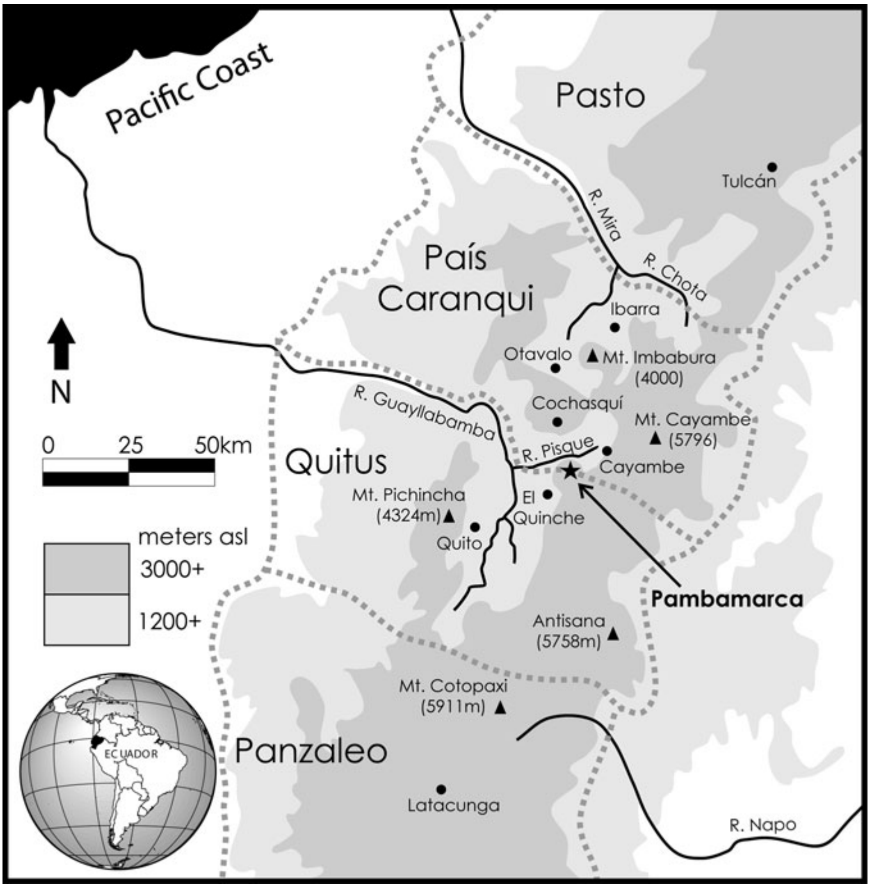

Figure 1. Regional map of northern Ecuador that includes locations mentioned in the text and the debated names of ethnic groups. Pambamarca is located along the proposed border between the peoples of Quito and the País Caranqui to the north (Map by C. Gifford).

and extensive preexisting trade routes with access to exotic goods (Alchon 1991:8; Salomon 1986).

According to chroniclers, most people on the northern frontier did not welcome the Inka: the País Caranqui groups banded together and ferociously resisted the Inka for a decade or longer (Betanzos 1987[1551–1557]; Cabello Valboa 1951[1586]; Cieza de León 1959[1553]; Cobo 1979[1653]; de la Vega 1961; Montesinos 2010; Sarmiento de Gamboa 2007 [1572]). We believe these groups were well positioned to offer stiff resistance because intraregional struggles were already part of the narrative (see Arkush 2011 on the Colla). These groups had been in "balanced opposition": a power dynamic in which local feuds could be easily dropped in favor of confederations bound together to confront imperial invaders. The weakly integrated and decentralized País Caranqui may have been stronger precisely because of these heterarchical coalitions. Describing his concept of the tribal imagination, Fox (2011:78) contextualizes Arab resistance to western empires in terms of the Arab proverb, "I against my brother; my brother and I against my cousins; my cousins and I and my brothers against the world." He argues that tribal states are, in a sense, the default form of social organization, providing the social glue necessary to make a sustained defense against larger complex imperial bureaucracies.

When the Inka arrived, Cieza de León noted, "Those of Otavalo, Cayambe, Cochasquí, Pifo, and other peoples north of Quito had made an alliance among themselves not to allow themselves to be dominated by the Inca" (1959

Downloaded from https://www.cambridge.org/core. University of New England, on 28 Mar 2019 at 21:59:08, subject to the Cambridge Core terms of use, available at https://www.cambridge.org/core/terms. https://doi.org/10.1017/laq.2018.80

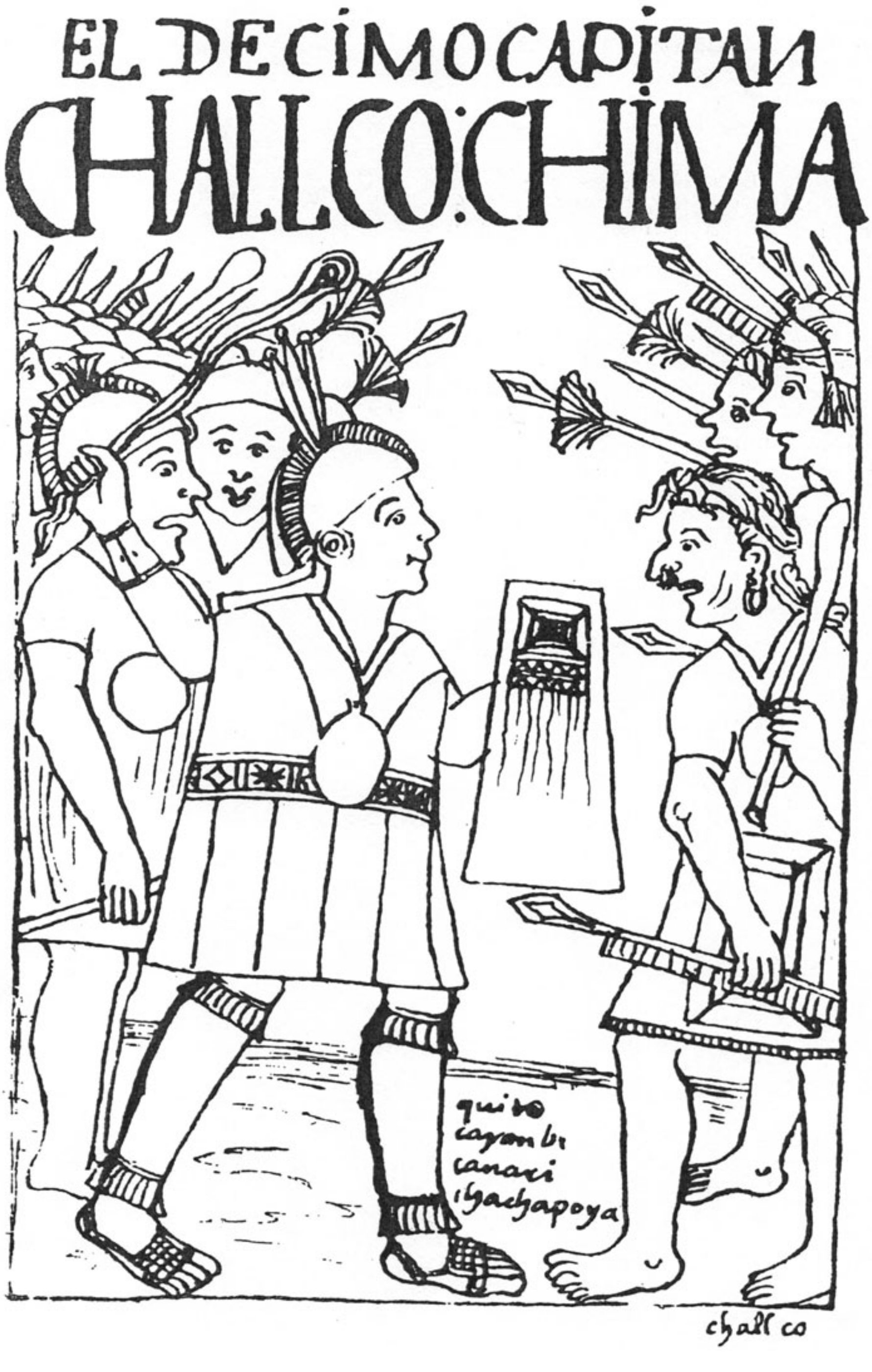

Figure 2. ***El décimo capitán*** **by Guaman Poma de Ayala (1980:140). The Cayanbi (Cayambe) are noted here as an enemy. The Inka soldier is swinging a slingshot, a common weapon from our excavations. Note the expression of fear on the faces of these Inka warriors.**

[1553]:48); Cabello Valboa corroborated this account (1951[1586]:369). Guaman Poma de Ayala (1980:140) memorialized the resistance in a drawing depicting the gruesome visages of the indigenous Ecuadorian populations engaging in a pitched battle against the tenth Inka captain

Downloaded from https://www.cambridge.org/core. University of New England, on 28 Mar 2019 at 21:59:08, subject to the Cambridge Core terms of use, available at https://www.cambridge.org/core/terms. https://doi.org/10.1017/laq.2018.80

Challco-Chima (Figure 2). After initial setbacks, the Inka regrouped and sent mitimaes from southern Ecuador to construct fortresses along the frontier. Additional battles were fought over a period spanning at least a decade. In one battle Huayna Capac was almost killed at one of the Cayambe fortresses (Cobo 1979[1653]:157; see also Betanzos 1996[1557]; Sarmiento de Gamboa 1960[1572], 2007[1572]). Later, the Inka, using reinforcements from Cuzco and Tomebamba, tricked the indigenous soldiers into leaving their fortresses and deployed a surprise attack that ended the resistance. Twenty to thirty thousand País Caranqui soldiers then fled to Yaguarcocha (the Lake of Blood) near modern-day Ibarra where every able-bodied male was supposedly slaughtered (Cieza de León 1959 [1553]; Cobo 1979[1653]; Sarmiento de Gamboa 2007[1572]). Once victorious, the Inka continued northward into the Pasto territory of modern Colombia and ruled the area until the arrival of the Spanish.

The Pambamarca Complex

Located about 40 km east–northeast of Quito in the Cayambe Province, the Pambamarca complex consists of 18 installations spaced a kilometer or more apart on top of and around the ridges and peaks of the extinct Pambamarca stratovolcano (Figure 3; Table 1). Two basic environmental zones define the 150 km^2 area of Pambamarca; the upland *páramo* above 3,000 m asl where 15 sites are found and the lower

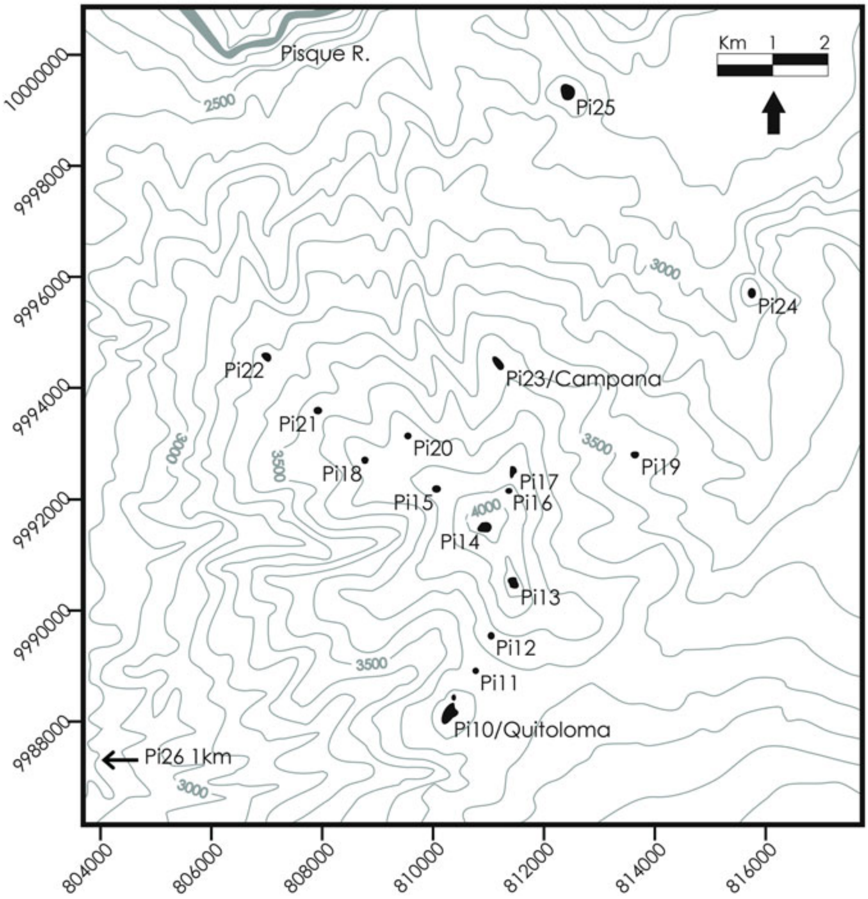

Figure 3. Topographic map of the Pambamarca complex. Pi26 in El Quinche lies just off the map to the southwest, and the highest fortress, Cangahua Pucara is farther up the ridge line on the continental divide to the southeast (Map by S. Connell and C. Gifford).

Downloaded from https://www.cambridge.org/core. University of New England, on 28 Mar 2019 at 21:59:08, subject to the Cambridge Core terms of use, available at https://www.cambridge.org/core/terms. https://doi.org/10.1017/laq.2018.80

Table 1. Archaeological Sites of the Pambamarca Complex.

Common Name	Name on 1:50,000 Map	Site Type	Elevation (m asl)	Interior surface area (m2)	Plaza Schuller 1977	Hyslop 1990	Environmental Zone
Quitoloma	Quito Loma	I	3,800	1,09,000	Pi10	5	Paramo
El Sombrero	Las Coles	II	3,720	5,027	Pi11	4	Paramo
Janchirumi	Las Coles	II	3,800	25,447	Pi12	3	Paramo
Jambimachi	Cerro Jambi Machy	II	4,078	15,394	Pi13	2	Paramo
Pambamarca/ Frances Urco	Cerro Pambamarca	I	4,075	1,01,788	Pi14	1	Paramo
Pinan	Loma Cunotola	II	3,896	28,353	Pi15	6	Paramo
Pi-16	Not identified	II	3,930	31,416	Pi16	11	Paramo
Celda	Loma de Toaquiza	I	3,892	66,052	Pi17	12	Paramo
TablaRumi	Tabla Rumi	I	3,791	38,013	Pi18	8	Paramo
Pucara	Loma Pucarito	I	3,480	53,093	Pi19	14	Paramo
Censo	Loma Censo Pucara	I	3,796	49,087	Pi20	7	Paramo
Chiripampa	Cerepamba	II	3,600	9,503	Pi21	9	Paramo
Achupallas	Cerro Achupallas	I	3,400	57,256	Pi22	10	Paramo
Campana	Loma Campana Pucara	I	3,614	61,575	Pi23	13	Paramo
Pucarito	Loma Pichimbiro	III	3,020	96,211	Pi24	n/a	Temperate
Pingulmi	Guana Loma	III	2,997	1,24,410	Pi25	n/a	Temperate
Pucara El Quinche	Not identified	III	2,813	unknown	Pi26	n/a	Temperate
Cangahua Pucara	Cangahua Pucara	I	4,178	unknown	n/a	n/a	Paramo

temperate zone below 3,000 m asl where 3 sites are found.

The Pambamarca Archaeology Project (*Proyecto Arqueológico Pambamarca*) began under the auspices of the Instituto Nacional de Patrimonio Cultural del Ecuador (INPC) with a goal of determining who built and used the sites, given that historic sources, chroniclers, colonial *visitas*, and even modern lore disagree on that subject. Chronicler accounts often referred to these installations as forts or *pucaras*, but attributed them to both Inka and indigenous builders, with no details given to differentiate the two (Cieza de León 1959[1553]:47; Cobo 1979[1653]:157; Espinosa Soriano 1988; Ramón Valarezo 1987; Salomon 1986; Sarmiento de Gamboa 2007[1572]:141–144). For example, Cieza de León first noted, “Knowing of his (Huayna Capac’s) presence in Quito… they (the indigenous) had built forts and stockades,” and then a page later wrote this: “The Inka and his army came as far as this region (north of Quito), and there he ordered stockades and fortresses, which they call *pucaras*” (1959 [1553]:47, 48). These accounts thus have proven to be useful but problematic tools, because many contain inherent colonial biases, Eurocentric descriptions, and chronological discrepancies (Julien 1993, 2000; Morris 1982; Rowe 1945, 1947; Stanish 2001). Part of the ambiguity noted in accounts about Pambamarca may also stem from a lack of understanding by the Spanish about what the term *pucara* actually encompassed, or bias may have existed regarding the features and functions that fortresses “should” have (Keeley et al. 2007:56). Possibly the term “fortress” may be too limiting for the Pambamarca sites and many others within precontact South America (Arkush 2011).

Previous archaeological work in the northern Ecuadorian highlands began with site recording by Squier (1877), Uhle (1926, 1930, 1939, 1960[1925]), and Jijón y Caamaño (1914, 1952). Pre-Inka Late Period sites are characterized by earthen hemispherical mounds (*tolas*) and massive truncated platform mounds, some with ramps hundreds of meters long. Although many mounds have been destroyed, evidence shows pre-Inka centers were located near Ibarra, Otavalo, Zuleta, Cochasquí, Cayambe, and El Quinche (Figure 1), with the last two sites being the closest to our study area (Cordero Ramos 1998; Jijón y Caamaño 1914). Survey and excavations were also completed in the Pambamarca complex (Fresco et al. 1990; Hyslop 1990; Oberem et al. 1969; Plaza Schuller 1976,

Downloaded from https://www.cambridge.org/core. University of New England, on 28 Mar 2019 at 21:59:08, subject to the Cambridge Core terms of use, available at https://www.cambridge.org/core/terms. https://doi.org/10.1017/laq.2018.80

1977, 1978) and are discussed in detail later in the article.

Archaeological Survey

Initially funded by an NSF High Risk Grant (BCS-0331866), our work began with three survey phases: (1) ground-truthing existing site maps and remapping them if necessary, (2) locating previously unrecorded features, and (3) using geophysical techniques to look for early construction phases. We produced detailed site maps for each of the 18 sites (Figure 4; see Plaza Schuller 1977) and identified 3 site types (Table 1).

Type I. Nine sites found in the *páramo* at an average elevation of 3,780 m asl are large installations all constructed of masonry stones that were mined from the immediate location and averaged 6.70 ha in area (excluding the partially mapped and largest, Cangahua Pucara; Table 1). These *pucaras* contain typical defensive features, such as multiple high concentric stone walls around a hilltop or ridge averaging 2–5 m in height, with deep fosses and adjacent parapets (noted in Keeley et al. 2007). At least three Type I sites and one Type II site are connected by deep trenches measuring 1 m wide and 1–2 m deep that could be used to move soldiers quickly and safely between sites (Figure 4). Within the sites, entryways are baffled or tightly controlled or lead into open spaces that either contain numerous well-preserved stone structures, large boulders, or rock outcrops (interpreted as *huacas*) or nothing at all. Numerous rectangular house compounds, known as *kanchas*, contain two to four structures arranged around a patio. The typical structure size averages around 5 m^2; many of these structures are protected from east-to-west winds by their leeward location. A combination of magnetometry, resistivity, and ground-penetrating radar (GPR) at two Type I sites did not uncover any buried architecture nor did excavations reveal any pre-Inka occupation phases, suggesting a single phase of construction and abandonment (Sullivan 2007).

Most Type I enclosures have two significant Inka architectural features: an *ushnu* platform

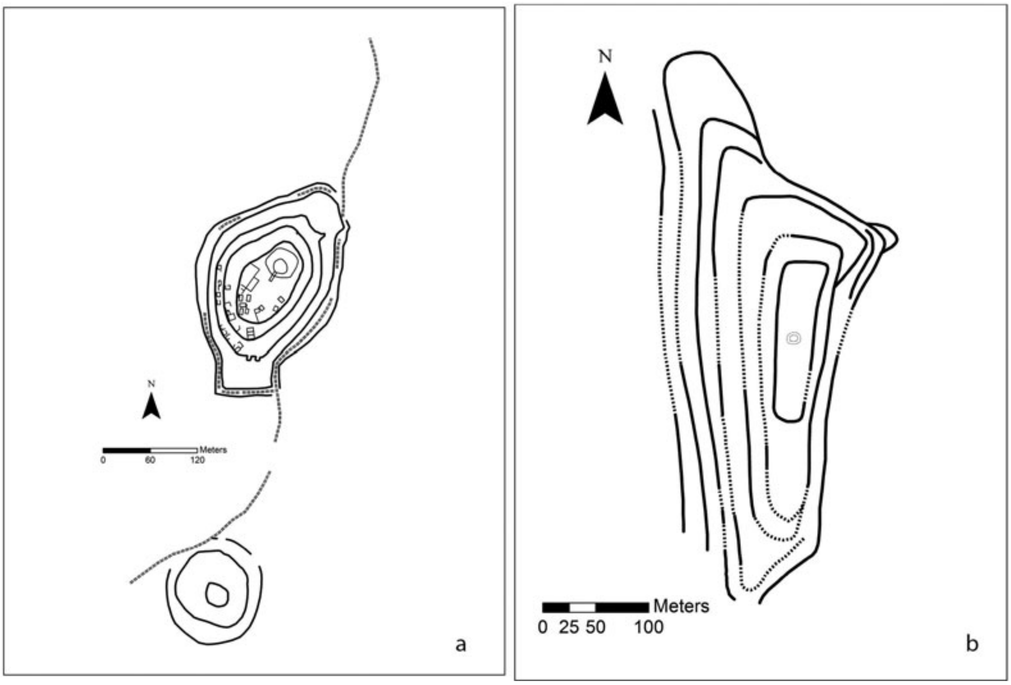

Figure 4. Survey maps of Type I, II, and III sites. (a) Pi17 Celda Pucara (Type I) and Pi16 (Type II) are connected by a sunken trench that runs through part of the fortress complex connecting the forts. (b) Pi24 Pucarito (Type III) is at 3,020 m asl in the temperate zone, with no standing structures and visible eroded architecture made of *cangagua* (Maps by C. Gifford and O. Wigmore).

Downloaded from https://www.cambridge.org/core. University of New England, on 28 Mar 2019 at 21:59:08, subject to the Cambridge Core terms of use, available at https://www.cambridge.org/core/terms. https://doi.org/10.1017/laq.2018.80

and a *kallanka*. *Ushnus*, strategically placed multitiered platforms, sometimes with a staircase, ramp, or shrine, were often found at the center of the site, in large open areas, or in main plazas. Although their exact function is debated (see Ramón 2017), they were thought to define sacred and political spaces (Hyslop 1990; Meddens 1997; Meddens et al. 2010; Meyers 2007; Moore 1996; Staller 2008). The second Inka feature is the *kallanka*, a long rectangular construction containing multiple entrances and sometimes gabled roofs (Hyslop 1990). Again, ambiguity surrounds this term, which may possible refer more to the structure's architectural form than its function. Most times *kallankas* are characterized as meeting halls or officer barracks, and although there is no consensus around a standardized *kallanka* size, they are much larger than other structures and are often located near features such as the *ushnu* and away from *kanchas* (Hyslop 1990).

Type II. Six high-elevation enclosures in the *páramo* (average elevation of 3,837 m asl) are smaller than the Type I installations, averaging 1.92 ha in area; they are defined by one or two low stone walls (less than 2 m high) surrounding central spaces containing few structures and few artifacts. Rather than being military in function, it appears that most Type II sites served ceremonial and/or economic functions within the Pambamarca complex (Anderson 2014).

Type III. Three large fortresses are found in the lower temperate zone at an average elevation of 2,943 m asl. These installations have an average size of 11.03 ha, are defined by high terrace walls and no visible standing architecture, and have suffered a high degree of damage from modern farming. Unlike Type I and II sites, a high density of living debris, including pottery, mammal bones, lithic and groundstone artifacts, was found on the surface. Excavations within the domestic terraces exposed local pre-Inka ceramic artifacts, sling stone caches, and the highest concentration of Inka-style pottery found at Pambamarca.

Architecturally, multiple construction phases are evident, suggesting continued use through time. Type III fortresses are composed of large shaped blocks mined from the local hard-packed, consolidated volcanic ash subsoil known as *cangagua*. Research from other sites in the País Caranqui (Athens 2003; Bray 1991; Cordero Ramos 1998; Currie 2001) characterizes this construction method as a local indigenous architectural style, suggesting the possibility that these enclosures were built by the Cayambes before Inka contact or during the imperial process. A survey completed between the Type III sites showed the existence of previous and contemporaneous sites, and future research is needed to determine how they fit into the imperial narrative of Pambamarca.

Archaeological Excavations at Type I Sites: Quitoloma and Campana Pucara

Although all site types are vitally important to the holistic narrative of imperial formation at Pambamarca, our analysis in this article focuses on excavations at two Type I sites, Quitoloma (Pi10) and Campana Pucara (Pi23). Archaeology sites in the Pichincha province of Ecuador are given the designation Pi and a number, although we use site names recorded in historic records or noted by local communities where possible (Table 1).

Quitoloma (Pi10)

Characterized by high concentric walls with deep exterior ditches draped over a large hill (Figures 5 and 6), Quitoloma marks the southern extent of the Pambamarca complex and offers views south toward Quito. Initially investigated by Oberem and colleagues (1969), Quitoloma measures 450 m long by 280 m wide: the 10.9 ha area is divided by interior walls into seven spaces that contain structures, exposed bedrock outcrops, solitary boulders, a natural water source, and open sections devoid of settlement (Figure 5). Based on survey and five test excavations, Oberem determined that Quitoloma was an Inka fortress built to conquer the Cayambe. Excavations of one circular structure (S1), three rectangular structures (S2–4), and a unit in the open area to the south (S5) recovered surprisingly strong signatures of Inka life, including *aribalo* fragments, a flute, a spear point, spindle whorls, obsidian, and bola stones (Oberem et al. 1969). Our research expanded on Oberem's work to investigate additional residential

Downloaded from https://www.cambridge.org/core. University of New England, on 28 Mar 2019 at 21:59:08, subject to the Cambridge Core terms of use, available at https://www.cambridge.org/core/terms. https://doi.org/10.1017/laq.2018.80

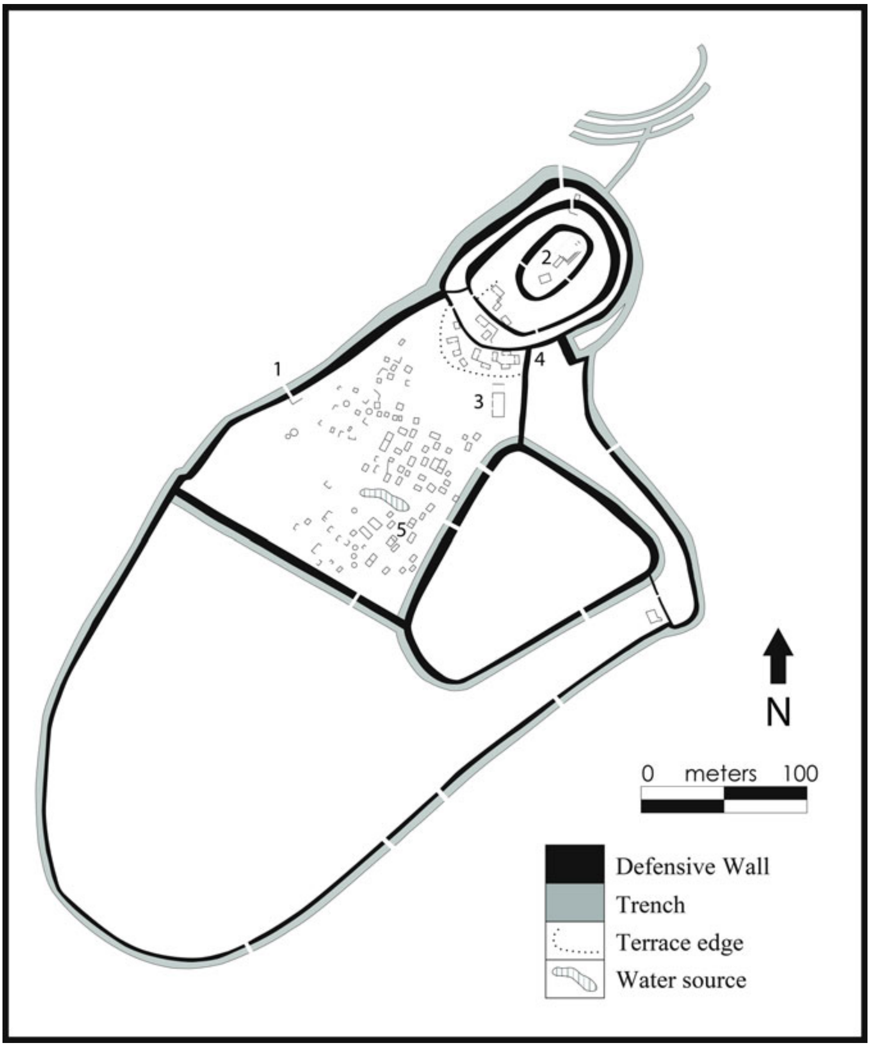

Figure 5. Quitoloma survey map with points from the text labeled (1) entranceway location, (2) *ushnu*, (3) *kallanka*, (4) *kanchas* or patio groups, and (5) armory or munitions storehouse (Map by C. Gifford).

structures, the *ushnu*, *kallanka*, entranceways, and more open spaces. We also conducted a systematic survey of plowed fields outside the main walls, finding no evidence of structures or occupation other than the Inka roads discussed later (Sistrunk 2010).

On the north end, Quitoloma is defined by three enclosed interior areas with standing architecture, contained by three successive concentric walls (Figures 5 and 6a). The incomplete defensive ditches and walls suggest abandonment of the site or termination of its need. To the south lies a partially enclosed section with *kanchas* abutting an open space containing the *kallanka,* after which a buffer zone exists before the main residential portion farther south. This section contains 104 mapped structures in patio groups, bisected by main avenues, alleys, or the water source; many have interconnected walls, suggesting densely packed living quarters (Figure 5). Closer to the *kallanka*, fewer residential structures are found, and their size (6 x 3 m in the interior; Figure 6b) differs markedly from that of room blocks located in the south (3 x 2 m in the interior, label #5 in Figure 5). The differences in structure size may indicate that high-status

Downloaded from https://www.cambridge.org/core. University of New England, on 28 Mar 2019 at 21:59:08, subject to the Cambridge Core terms of use, available at https://www.cambridge.org/core/terms. https://doi.org/10.1017/laq.2018.80

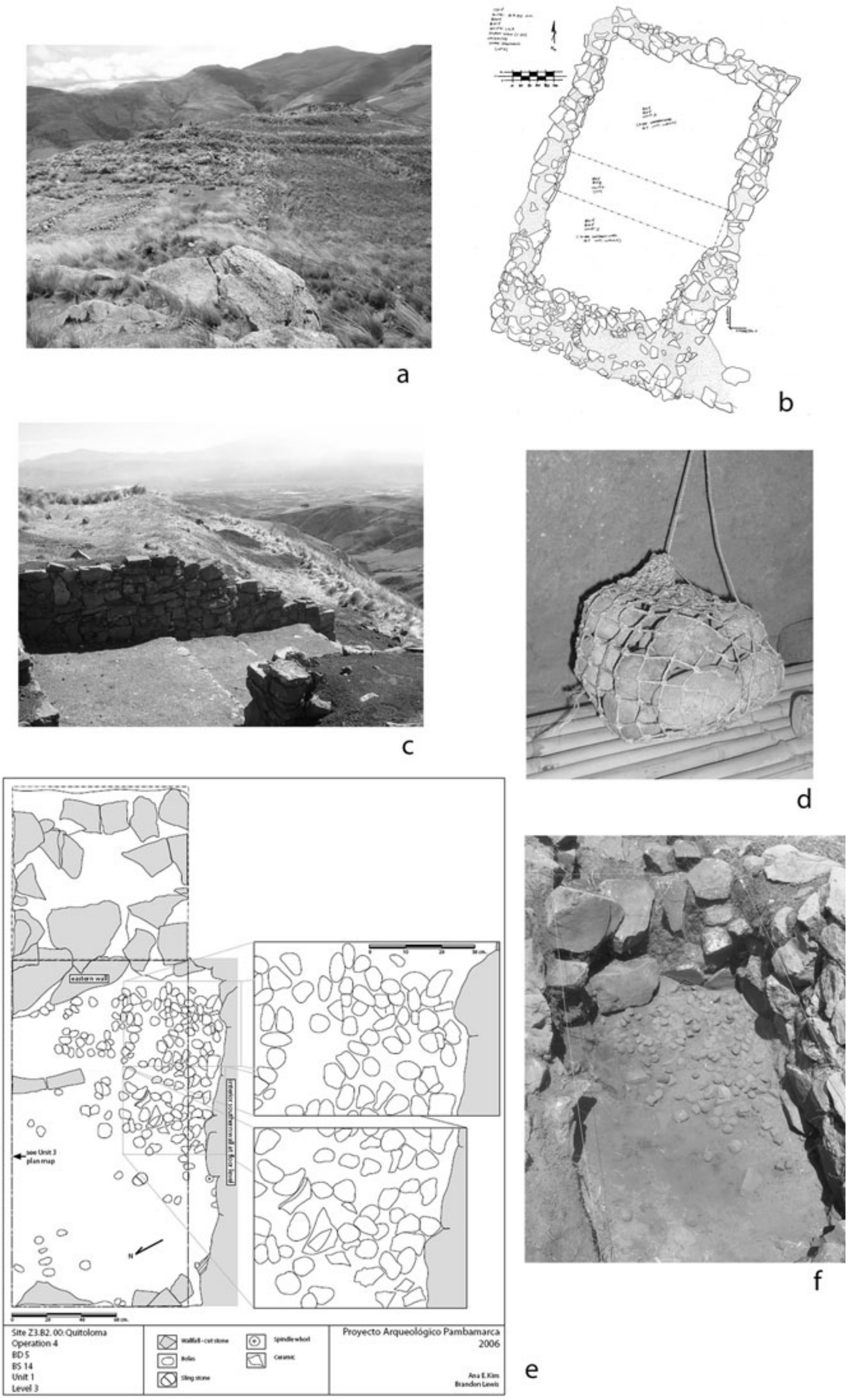

Figure 6. Quitoloma research photographs and plan drawings: (a) looking north from the middle of the site with the *kallanka* in the foreground to the left, the *ushnu* in the middle right, and along the distant ridge to Pambamarca pucara (Pi14); (b) plan drawing of a typical room block, marked #4 on Figure 5; (c) entranceway reconsolidation completed by Rudy Larios; (d) example of sling stone war bag from the Cochasquí site museum; (e) and (f) sling stone deposit or ammunition dump in Structure 14 interpreted as an armory or weapons storehouse (Photographs by D. Morin, S. Connell, and C. Gifford; drawings by Lucas Johnson, Ana Kim, and Brandon Lewis).

Downloaded from https://www.cambridge.org/core. University of New England, on 28 Mar 2019 at 21:59:08, subject to the Cambridge Core terms of use, available at https://www.cambridge.org/core/terms. https://doi.org/10.1017/laq.2018.80

Inka lived in the north end, separated from the other soldiers.

To the south and east of the main living area there are walled-off empty spaces, some of which contain several large boulders thought to be *huacas*. Excavations by Oberem and colleagues (1969) at the southernmost point recovered a cache of Inka-style ceramic artifacts associated with a prominent rock formation. Testing by our team in these empty areas produced no artifacts, but it is possible that the area was used for herding, military practice, or activities associated with *huacas*.

A total of seven entrances are found in the outer walls. A single doorway on the northeast side offers the easiest point of access, but leads to a deep, walled-off dead space, perhaps constructed to lure combatants into a passageway designed for ambush. To the north a restricted entrance with a guard post leads to the inner areas. Four simple entrances are found on the southeast side, likely designed to allow warriors to stream out and overwhelm an opponent. Access to the main interior occurs via an entrance to the west (label #1 on Figure 5). The locale was excavated in some detail by Antonio Fresco and colleagues (1990) and then reexposed in 2003 by Rudy Larios to reconsolidate and preserve the stairway (Figure 6c). This entrance to the west was heavily guarded: masonry walls extend north and south from the lower steps reaching 3 m in height. At the top of the stairwell a 1 m thick freestanding wall abuts the northeast corner, forcing entrants to turn left for site access. Inside the entranceway an embrasure was built to hide a sentry, and nearby a dense collection of more than 50 sling stones was recovered.

Quitoloma's Inka masonry style consists of roughly worked stone blocks carefully fit together with mortar and chinking stones, all of which can be mined at the site. Excavations showed no stratigraphic evidence of earlier construction or occupation, indicating that Quitoloma was constructed in a single phase and not over an indigenous settlement. Exposed charred material from construction fill at the interface with sterile soil (near #4 on Figure 5) was radiocarbon dated to 330 +/- 40 BP (Beta-240835; $\delta^{13}C = -23.71\%$) or 1450–1650 cal AD (calibrated at 2σ with the program IntCal04), within the expected range for Inka construction and occupation. The calibration curve for northern Ecuador extends date ranges, yielding the misleading continuation of the date into Spanish colonial times.

Quitoloma's *ushnu* is located at the north end in a small enclosed plaza (label #2 in Figure 5), with two terrace levels and a central staircase leading down its south side. The quadrilateral mound, measuring 12 m x 16 m and 2.7 m high, was heavily looted, and salvage work within found no previous occupation layers. Access to the *ushnu* tier was possible via two 1 m wide doorways on the east and west sides. Small amounts of Inka ceramic vessels were found along the basal steps of the *ushnu* and alongside low walls of a structure in the plaza to the south.

In 2005 excavations also occurred at the *kallanka,* a large rectangular structure measuring 14 m x 7 m located in the central plaza area (label #3 in Figure 5). Initially identified as the Inka *casa comunal* by Oberem and colleagues (1969), the building had two doorways on the west side. GPR survey and excavations showed that a smaller adjoining room on the north side was added later (Sullivan 2007). Two trenches across the *kallanka's* center produced a handful of Inka pottery forms such as *arribalos* (Form 1), one footed *ollas* (Form 10), and shallow dishes (Form 13; Bray 1991: Figure 7.5; Meyers 1981); a third trench exposed low masonry walls.

Our work within the residential area focused on expanding on what Oberem had recovered in an effort to better define the occupants of the site, use of the site, and length of occupation. We chose to excavate 12 rectangular structures and patio areas from across the site. All contained evidence indicative of daily activities, such as food preparation and weaving, with finds including different cooking and serving vessels, groundstone fragments, spindle whorls, faunal remains, and other artifacts associated with the mundane. These utilitarian findings do not occur in dense concentrations, indicating a shorter-term occupation, but they do provide evidence that families accompanied soldiers on their deployments. Several *kanchas* appear to have expanded as the number of occupants rose. For example, excavations at the north and west structures of a patio group nearest to the *kallanka*

Downloaded from https://www.cambridge.org/core. University of New England, on 28 Mar 2019 at 21:59:08, subject to the Cambridge Core terms of use, available at https://www.cambridge.org/core/terms. https://doi.org/10.1017/laq.2018.80

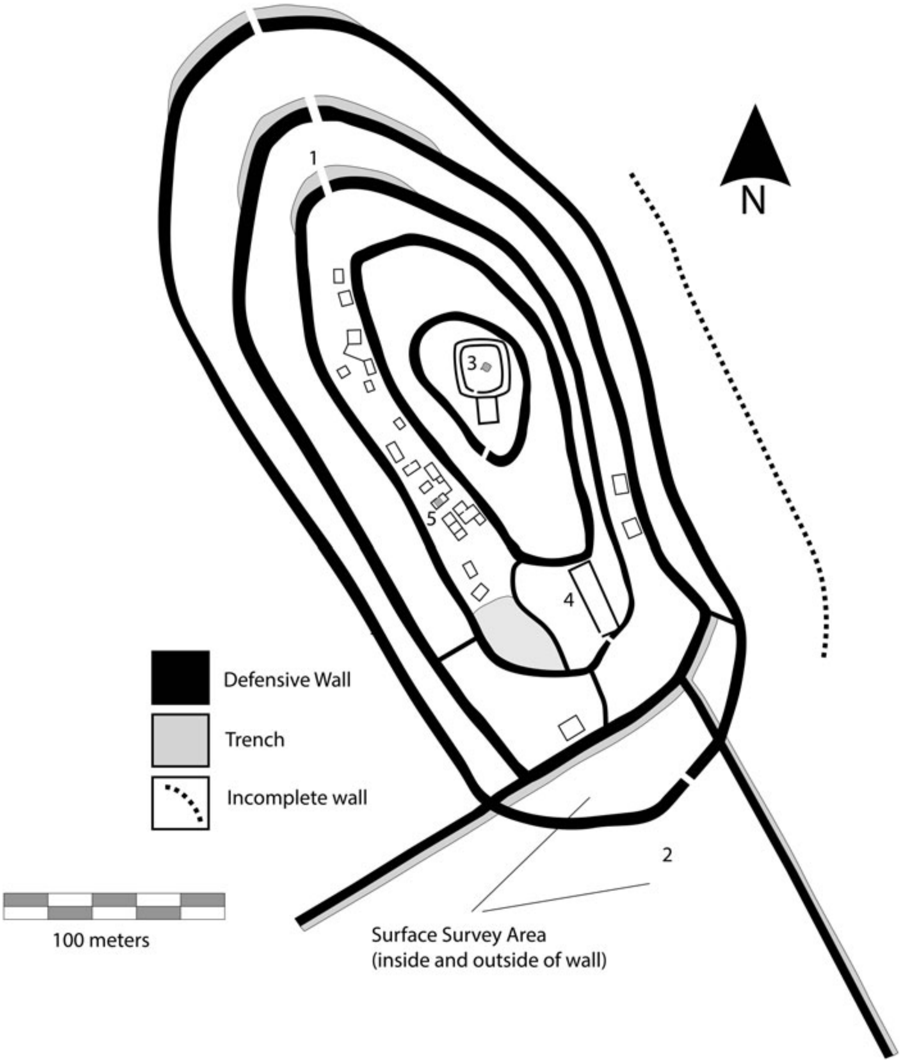

Figure 7. Campana Pucara survey map with points from the text labeled (1) entranceway and exterior wall excavations; (2) survey area for sling stones inside and outside of walls; (3) *ushnu*; (4) *kallanka* and area of GPR survey; and (5) room block excavations (Map by C. Gifford).

(label #3 in Figure 5) uncovered secondary filler walls abutting the original structures, creating a kitchen space with dense amounts of burned animal bone and cooking vessel fragments.

By far the most common artifact recovered within every residential structure excavated at Pambamarca was the sling stone. At many Andean fortified sites, sling stones tend to dominate because they are usually readily available and transportable (Topic 1989). In Quitoloma structures, they were often found in stacks of seven or more placed directly inside the doorway and in association with small obsidian blade fragments and a single nondiagnostic sherd (see the later discussion of the example from Campana Pucara). The consistency of this pattern suggests that warriors may have traveled with pouches (like a Scottish sporran) that contained these items. The broken sherd could provide extra support and strength to the bottom of the bag, and the obsidian blade fragments could have been inserted into clubs or mace heads. This interpretation of Inka "war bags" is supported by the discovery of a bag by Max

Downloaded from https://www.cambridge.org/core. University of New England, on 28 Mar 2019 at 21:59:08, subject to the Cambridge Core terms of use, available at https://www.cambridge.org/core/terms. https://doi.org/10.1017/laq.2018.80

Uhle at Jaquí in the Yauca Valley, Peru, now located in the Hearst Museum (artifact number 4-8306). Another example is on display at the nearby Cochasquí site museum (Figure 6d).

The most obvious example of militarism at Quitoloma was the discovery of an armory for ammunition storage. Excavations at Structure 14, in a patio group on the south side, exposed a top layer of pottery, a spindle whorl, a *boleador*, and a concentration of 171 sling stones in the southeast corner that was too dense to remove and likely covered still more ammunition beneath it (label #5 in Figure 5 and see Figure 6e and 6f). Charred material recovered from the living surface yielded a carbon date of 380 +/- 40 BP (Beta-240836; $\delta^{13}C = -23.21\%$) or 1440–1640 cal AD (calibrated at 2σ with the program IntCal04). It is puzzling that so much ammunition was left behind at the site. The occupants of Quitoloma may never have had the opportunity to go to battle, or the need to carry ammunition lessened as the Inka moved northward. It also may have been common practice to leave everything behind when the soldiers went home, or perhaps Quitoloma was left stocked in case of future needs, befitting a defense in-depth strategy (D'Altroy 1992). Some chroniclers suggest the forts in Cayambe were still occupied during the reign of Atahualpa before the arrival of the Spanish (Betanzos 1996[1557]:200). Whatever the reasons, Quitoloma continues to exist in an abandoned state of readiness.

Campana Pucara (Pi23)

Investigations at Pambamarca also focused on Campana Pucara primarily because of its excellent preservation and critical position as the northernmost Type I site (Figures 3 and 7). Campana Pucara was a strategically important locale for rebuffing enemy attacks and for maintaining vigilant oversight of troop movements because it overlooked the Cayambe basin and would have been the initial line of defense (Figure 8a). When compared to Quitoloma, the material record of the site is better preserved and contains similar, but more accessible, architectural patterning. The site is defined by five concentric walls divided into sections containing 23 structures on the leeward side below the ridge top (Figure 7). There are three aligned entrances on the north side and a single entrance to the south that link Campana Pucara by a trench to Pi17, Pi16, and Pi14 (Figures 3 and 4). Several of the outer walls are associated with defensive features: deep ditches and parapets. The absence of pre-Inka occupation phases, coupled with the presence of partially worked stone blocks, an *ushnu* with a ramp, a *kallanka*, several *kanchas*, and high counts of weaponry and Inka style pottery, suggests that the site is Inka (Figure 7).

Our initial excavations were made along a northern inner defensive wall (label #1 in Figure 7) and a nearby looter's trench (Figure 8b) and were directed to identifying wall construction techniques and the existence and/or depth of a floor. They revealed that the wall was faced with large stones on each side, and a fill (uniform in color, content, and texture) was added, indicating it was erected in a single construction episode. An orange clay cap rested on top of the fill inside the wall, which may have (1) provided a hard surface between the uppermost courses of stones used to house sling stones or bolas and (2) served as a type of mortar that helped hold the wall together. Two compacted floors were recovered that abutted to the lowest course of stones on the inside face of the defensive wall. The older flat floor was connected to the lowest course of stones, and ceramic vessels from the floor surface showed evidence of burning. A sloped or ramped ephemeral second floor was added later, which likely served as a banquette to elevate interior combatants above the wall to better launch sling stones. It is possible that other walls on the north side have similar defensive features, especially given that many are lined with northern ditches that make access difficult.

Findings from additional excavations support the militarized nature of the site. Seven excavation units placed in residential structures on the western side produced caches of sling stones (label #5 in Figure 7). These were usually stockpiled near entrances, bastions, parapets, outer walls, or within structures, and the size, quantity, and placement of the stones are identical to those uncovered at Quitoloma (Figure 8c). Although we did not find a weapons storehouse, it is possible it remains hidden in an unexcavated

Downloaded from https://www.cambridge.org/core. University of New England, on 28 Mar 2019 at 21:59:08, subject to the Cambridge Core terms of use, available at https://www.cambridge.org/core/terms. https://doi.org/10.1017/laq.2018.80

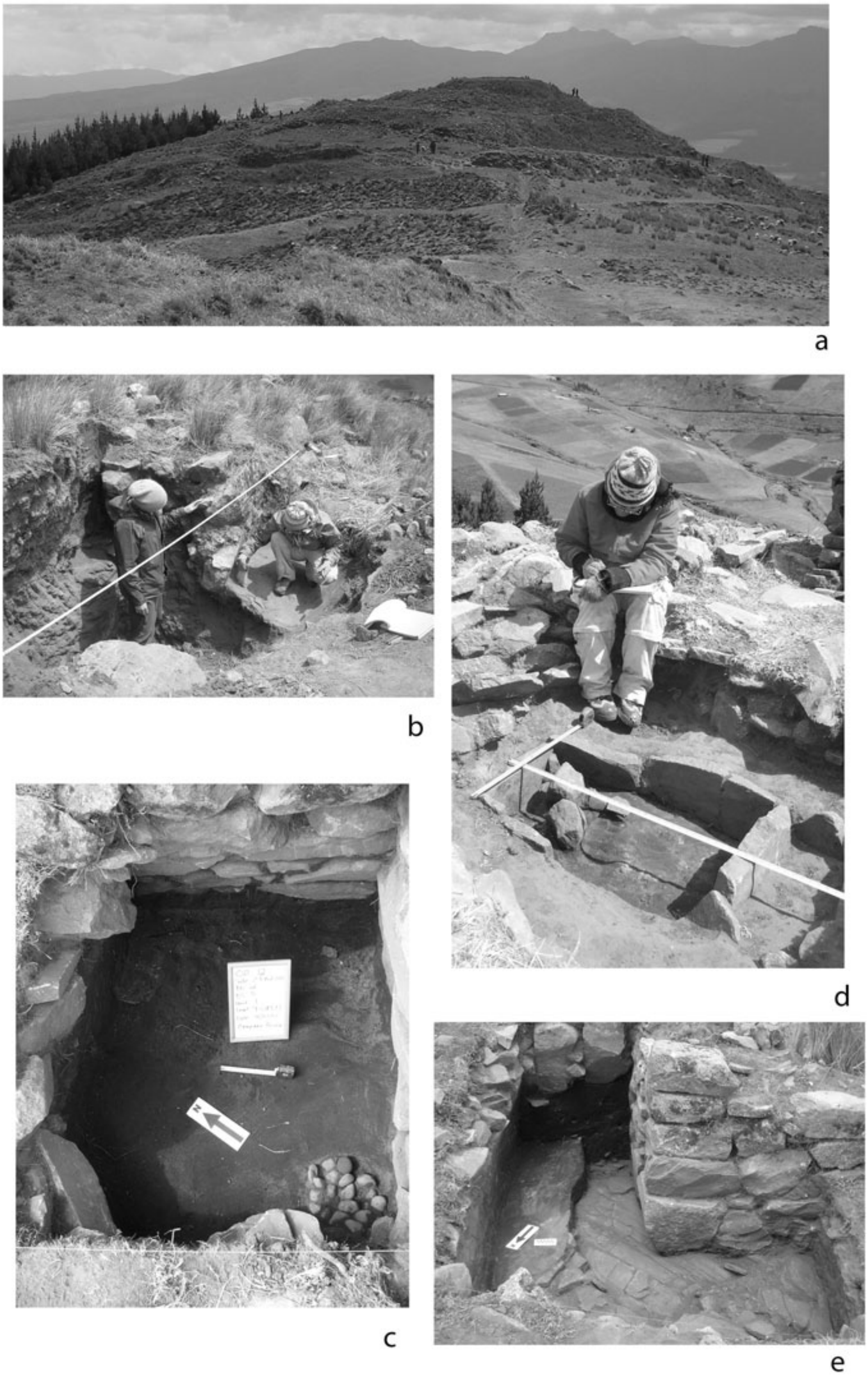

Figure 8. Campana Pucara research photographs: (a) Looking north into the main entranceway from the south, note the plowed areas that were surveyed, which have sling stones in the foreground field and none in the background field. People are standing at the *kallanka* inside the main gate; (b) students are cleaning the looter's trench, exposing wall construction and a tamped rampart sloping off the wall; (c) sling stone cache is found inside a residential structure; (d) documentation of the stone-lined box in the area marked #5 in Figure 7; (e) layer of charred material below the architecture marked by a north arrow. The standing Inka construction on the right cuts into the deposit, and a later wall addition is above the burned deposit at the back of the unit. Photographs by A. Anderson, S. Connell, and C. Gifford.

Downloaded from https://www.cambridge.org/core. University of New England, on 28 Mar 2019 at 21:59:08, subject to the Cambridge Core terms of use, available at https://www.cambridge.org/core/terms. https://doi.org/10.1017/laq.2018.80

structure. Not only were sling stones recovered in situ but they were also found outside the site during a salvage survey. Although the highland sites tend to have incurred less farming damage than the lowland sites, part of the southern perimeter wall was plowed between field seasons, which provided an opportunity to conduct a surface survey both on the interior and exterior of the wall (Figure 7, label #2, and Figure 8a). The difference in the collections from each area is striking. The interior collection was dominated by Inka ceramic forms and lithic artifacts, whereas the exterior collection had far more sling stones than other artifacts. Furthermore, these sling stones were found clustered toward the southern end of the plowed field, leaving a noticeable gap free of sling stones closer to the circuit wall, indicating that these stones were slung from inside toward groups on the outside (and not, for instance, slung toward the walls from outsiders). These findings are therefore some of the project's clearest evidence for possible acts of violence within Pambamarca.

Similar to Quitoloma, the cleaning of a deep looter's trench within the *ushnu* showed that it was built in a single phase (label #3 in Figure 7). Attempts to locate earlier construction phases were also made using a geophysical survey within the main plaza adjacent to and overlapping the *kallanka* (label #4 in Figure 7): it identified no subsurface architectural features. A combination of GPR, magnetometry, and resistivity survey did demarcate the *kallanka* limits, defined by its large rectangular shape and the presence of three doorways in the west wall. Four excavation units in the southern end revealed that the wall's fill contained embedded carbon deposits radiocarbon dated to the Inka Period. The conventional radiocarbon age for collected charred material is 340 +/- 40 BP (Beta-241879; $\delta^{13}C = -23.9\%$) or 1450–1650 cal AD (calibrated at 2σ with the program IntCal04). Evidence from the area west of the *kallanka* suggests that an outside plaza abutted the second-lowest course of the *kallanka* wall. In contrast to the Quitoloma pottery, an excellent collection of finely decorated pieces and discarded Inka ceramic artifacts, including Forms 1, 10, and 13, were found in a small alleyway between the *kallanka* and the outer defensive wall to the south. Although ceramic artifacts dominate the collection, we also found lithic artifacts and sling stones; however, the quantity of munitions recovered here was far lower than in residential parts of the site.

We also conducted research at five stone structures and one patio area within the western residential sector of the site (label #5 in Figure 7). The structures, measuring between 2.5 m x 4 m to 5 m x 5 m, contained Inka and local País Caranqui ceramic wares, ammunition, spindle whorls, and lithic artifacts, suggesting that a variety of activities occurred within these buildings. Several of the structures had two living floors. Older floors extended beneath standing walls and were associated with burned artifacts and ash deposits, a sample of which yielded a date of 340 +/- 40 BP (Beta-240838; $\delta^{13}C = -22.0\%$) or 1450–1650 cal AD (calibrated at 2σ with the program IntCal04). One structure contained a sunken stone-lined box filled with carbonaceous material, which was perhaps used for heating the room or cooking food (Figure 8d). Evidence of charred material from its last use dates to 400 +/- 40 BP (Beta-241878; $\delta^{13}C = -22.9\%$) or 1430–1530 cal AD, 1560–1630 cal AD (calibrated at 2σ with the program IntCal04), and 1440–1490 cal AD (1σ calibration with a single intercept). These dates, like those from Quitoloma, coincide with activities primarily taking place during the transition from the Late Period to the Inka Period.

One anomalous feature was a sample of charred material either from a collapsed thatched roof or an *estera* (woven floor mat), which was found directly on the bedrock and below the architecture; it was associated with an extensive ash layer containing calcined bone, carbon, Late Period and Inka Period ceramic artifacts, burned soil, and extensive fibrous residue (Figure 8e). Our radiocarbon date was clearly pre-Inka Late Period, 550 +/- 40 BP (Beta-241839; $\delta^{13}C = -19.0\%$) or 1310–1360 cal AD or 1380–1440 cal AD (calibrated at 2σ with the program IntCal04). This isolated evidence for Late Period activity, although tantalizing, does not tie to any architectural construction, but it does suggest that some places on Pambamarca were used before the Inka arrived.

Based on the evidence recovered and presented here, it is possible to make a few summary

Downloaded from https://www.cambridge.org/core. University of New England, on 28 Mar 2019 at 21:59:08, subject to the Cambridge Core terms of use, available at https://www.cambridge.org/core/terms. https://doi.org/10.1017/laq.2018.80

statements about the nature of the construction and occupation of Campana Pucara. First, evidence shows this site was constructed quickly but thoroughly. For example, the large concentric defensive walls were constructed with field stones that were cut or worked to form well-fitted masonry faces and were erected in one building episode. Second, evidence has emerged suggesting later, less monumental building (or rebuilding) episodes. As noted, an additional floor was constructed along the interior of a northern defensive wall, and some residential areas had walls added later that were built over soil deposits containing artifacts. There is no evidence that the site was abandoned between these episodes nor that it was immediately occupied by a new group of people: the architecture and material culture remained uniform. The second moment of building may have happened rapidly after a brief disruption, signified by the presence of ash in most units. For the moment, it appears that this disruption was caused by a Cayambe attack, after which the site was repaired. Third, we can solidly state that material evidence shows the population living in Campana Pucara was well equipped for battle. Sling stones were recovered both inside residential structures in small caches, similar to those found at Quitoloma, and scattered in the field outside the outermost defensive wall. When this evidence is considered in conjunction with other overt military features of the settlement, we can confidently state this site was on the leading edge of the Inka military attack against the País Caranqui.

Discussion

The Inka imperial process was multivariate in practice, and the northern frontier of Tawantinsuyu was no exception. Earlier discussions of soft versus hard power, although useful, often simplify complex narratives of imperial domination and resistance (Alconini 2008; D'Altroy 1992; D'Altroy and Schreiber 2004; Hassig 1988; Khatchadourian 2016; Shimada 2015), and recent work for the Inka has moved past these dichotomies (Alconini 2016). Our research at Pambamarca, although far from complete, is beginning to flesh out these variations in the imperial narrative. With its three site types, work at Pambamarca provides an opportunity to examine the range of tendencies that groups undergo during imperial moments. Although not presented in detail here and awaiting further research, the Type II and III sites at Pambamarca suggest an Inka strategy of partial sovereignty with hegemonic control dictated by indirect rule, in which power was delegated to the local elites and reinforced through imperial material culture. These low-control, low-extraction resource procurement strategies in the provinces relied on reciprocal trade routes between Cuzco and places where local elites were established (Alconini 2008, 2016; D'Altroy 1992; D'Altroy and Schreiber 2004; Hassig 1988; Malpass and Alconini 2010). Under these conditions preexisting interaction spheres were consolidated, commerce was incorporated or altered, and little investment was put into military facilities or imperial infrastructure outside key locales, with peripheral centers being maintained by local elites and societies (D'Altroy 1992; Luttwak 1976).

Nevertheless, as Alconini (2016) and others have noted, Inka control strategies were frequently in flux based on their desired wants or needs from an area, especially along the frontiers. We argue that in the face of staunch resistance the Inka quickly implemented high-control, high-extraction resource strategies and constructed military installations exhibiting a form of direct rule. As elsewhere, monumental state architecture and imperial features, such as administrative centers, storage facilities, or agricultural terraces, were constructed, and the Inka co-opted local trade routes and resources, thereby affecting interregional exchange systems (Alconini 2008; Arkush 2011; Bram 1941; Bray 1991; Bray and Echeverría 2014; D'Altroy 1992; DeMarrais et al. 1996; Fresco 2004; Hyslop 1990; Malpass and Alconini 2010; Salomon 1986; Sistrunk 2010). For example, research in obsidian procurement at Pambamarca points to the use of specific resources by the Inka and their discontinued use by the Cayambe (Ogburn et al. 2009). This control was aided by the construction of the Inka roads found in Pambamarca. A stone-lined roadway climbs from El Quinche west of our study area and forks at the base of Quitoloma, with one path heading north toward

Downloaded from https://www.cambridge.org/core. University of New England, on 28 Mar 2019 at 21:59:08, subject to the Cambridge Core terms of use, available at https://www.cambridge.org/core/terms. https://doi.org/10.1017/laq.2018.80

Mt. Cayambe and another heading east toward Cangahua Pucara, a newly discovered *pucara* (Figure 3; Table 1). Located east of Quitoloma, the site is the highest in the region at 4,200 m asl, and it offers the broadest viewshed found at Pambamarca. Although Cangahua Pucara has not been tested, it is possible this locale was used by the Inka for command and control of movement through the area. This road heading east likely connects with a second road discovered near the village of Oyacachi (about 18 km away) that connects to the Quijos region, which sits at the upper reaches of the Amazon rainforest (Sistrunk 2010). The network of roads supports the premise the Inka were interested in controlling trade routes, which is strengthened by archaeological discoveries of Inka storehouses at Molino Loma adjacent to the Hacienda Guachalá north of Pi24 (Figure 3; see Fries 2010).

Although control of economic resources and movement was important, military strength was a key component of this imperial process, with efforts put toward physically controlling the region through fortress construction. More than 100 *pucaras* have been documented in northern Ecuador, with Pambamarca representing the highest concentration of fortifications (Alconini 2004; Bray 2015; Hyslop 1990; Lippi 1998; Plaza Schuller 1976, 1977). Research presented here describes our efforts to determine the nature of the Type I enclosures built in the high páramo, specifically to determine if there were features of direct control. We describe here two Type I sites, Pi10 Quitoloma and Pi23 Campana Pucara, and the evidence recovered shows both contained the following elements:

- architectural patterning indicative of an immediate defensive need
- room for, and evidence of, permanent garrisons used to secure a border and defend it
- weapons storage and individual munition deposits
- examples of attack, such as large-scale burning and subsequent rebuilding, or artifacts associated with an impending engagement

The upland sites were very well defended because of their location, multiple concentric walls, baffled or protected entryways, parapets, and guardhouses. Excavations revealed they were constructed in a single phase, with radiocarbon dates placing them solidly during the Inka-País Caranqui conflict, and Inka structural elements such as the *ushnu* and *kallanka* were discovered at each. Furthermore, weaponry is ubiquitous: caches of sling stones were found in most residential structures excavated, and an ammunition storehouse was discovered at Quitoloma, attesting to the military use of this site. The presence of utilitarian wares at both sites also indicates household activities and showed that people, even families, were permanently living within these walls for a period of time.

We suggest that the Inka thought of these enclosures as self-contained communities designed for the business of war. They are built in out-of-the-way locations with little opportunity for the occupants to exploit the surrounding environments, especially at Pambamarca where the Inka settled at higher elevations than the local sites. Of note, several survey transects between the Type I and II sites and the lower-elevation Type III sites did not produce any evidence of occupation, suggestive of an empty "no man's land" or buffer zone between the two zones (Figure 3). From up high the Inka could be masters of all below and import supplies from well-controlled areas to the south, while also establishing new routes of transport, such as the road to Oyacachi. In addition, they could run soldiers and supplies between fortresses using several trenches or pathways found connecting some of the sites (Figure 4). By the sheer number of Type I sites constructed at the Pambamarca complex, we can infer that there was a long-term standoff in which the Inka invested a great deal of resources to stabilize their military gains and continue to press northward to conquer the region.

Of special interest was evidence indicating an attack or struggle at Campana Pucara: sling stone artifacts recovered from outside the exterior walls indicated they had been thrown out of the fort. Additionally, at both Campana Pucara and Quitoloma there is strong continuity between artifacts and construction materials and methods, indicative of no change in the occupants, even though it is clear that they remodeled their homes and defenses. It does appear the sites

Downloaded from https://www.cambridge.org/core. University of New England, on 28 Mar 2019 at 21:59:08, subject to the Cambridge Core terms of use, available at https://www.cambridge.org/core/terms. https://doi.org/10.1017/laq.2018.80

were abandoned in a state of readiness, given the types, quantity, and quality of artifacts recovered. Although we cannot be certain when, why, or how the sites were abandoned, the lack of Spanish-era pottery does suggest they were not used when the Europeans arrived and thus were likely already abandoned by AD 1532.

We believe our data can help archaeologists understand the nature of empire. The evidence for a military encounter and the creation of a densely packed defensive installation supports our assertion that some of the Pambamarca sites were built in the face of staunch resistance, as told by the chroniclers. It is apparent the Inka invested a great deal of resources to extend their military gains and continue their expansion northward during this prolonged struggle. Subsequent research will flesh out other imperial tendencies that are sure to have been part of the complex imperial narrative of the northern frontier. We feel certain that behind this imperial moment we will uncover a local indigenous landscape of intraregional competition and warfare, one that explains additional dynamics of the imperial process.

Acknowledgments. First and foremost, the authors would like to thank the hundreds of students who contributed to the project, and we also owe a debt of gratitude to a long and diverse list of people. To begin with, we must acknowledge the major force behind the genesis of this research program, Don Carlos Pérez. In Quito, many thanks to the wonderful team of professionals at the Instituto Nacional del Patrimonio Cultural del Ecuador (permits granted in 2002–2006 have no numbers; 059-2007, 054-2009, 070-2011, 089-2012, and 059-2014 extended to 2015, 2016, and 073-2017) and at the Departamento de Arqueología represented by Fernando Mejía, Mónica Bolaños, Francisco Sánchez, Maria Moreira, and Rosillo Murillo. In Cangahua we are grateful to Padre Roberto Neppas, Elena Tandayamo, Elena Gualavasí, Maria Gualavasí, David and Mario Quimbiulco, Anibal and Rita Charro, and Franklin Imbaquingo. Also, we extend a very warm thank you to the Hacienda Guachalá and Diego Bonifaz, Gabriela Bonifaz, and Cristóbal Cobo. Thanks also to the members of the communities of Pambamarca, especially those in Chumillos Central and Melchor Farinango. Many scholars directly contributed and are always welcome to visit; they include Stephen Athens, Kathryn Maurer, Tamara Bray, Ernesto Salazar, David Brown, Florencio Delgado, Rudy Larios, Dennis Ogburn, Karen Stothert, Mariuxi Cordero, Manuel Coloma, Rudi Colloredo-Mansfield, Pepe Echeverría, Patty Mothes, Peter Hall, Ronald Lippi, Marc Becker, Larry Conyers, Brandon Lewis, and Alejandra Gudiño. We also owe a large debt of gratitude to the Ecuadorian students who have contributed to the project, including Julio Mena, Oscar Cajas, y Carlos Montalvo. We are grateful for the contributions of our home institutions—Foothill College, UCLA, and Columbia University—and their representatives. Our research has been supported partly by the National Science Foundation (BCS-0331866), the Fulbright Ecuador program, and the US Ambassadors Grant-in-Aid program, but primarily by our student field school program. Finally, to our staff and graduate students without whom we would have never been able to succeed in the work, thank you so very much for all your help. We are especially grateful for the assistance of these individuals: Mo Carpenter, Zev Cossin, Mary Sullivan, Vanessa Muros, Allison Loewen, Allison Gray, Angus Lyall, Morgan Hoke, Adriana and Andrea González, Siobhan Boyd, Ana Kim, Ollie Wigmore, Eric Fries, Eric Dyrdahl, Scotti Norman, Hannah Sistrunk, David Morín, Will Pratt, Ryan Hechler, Hannah Dibner, Brian Kranzler, Colin Jaramillo, Douglas Smit, Matt Golsch, Lucas Johnson, Valda Black, Sandy Schreyer, Ted Neff, Linda Neff, Kaitlin Yanchar, Angela Matusik, Matt Schauer, and Curtis Campaigne. Without them the work would never have come close to being completed. Thanks for teaching, helping, and listening.

Data Availability Statement. A complete digital copy of the data presented in this article is available on request from the author: connellsamuel@foothill.edu.

References Cited

Alchon, Suzanne Austin
1991 *Native Society and Disease in Colonial Ecuador*. Cambridge University Press, New York.

Alconini, Sonia
2004 The Southeastern Inka Frontier against the Chiriguanos: Structure and Dynamics of the Inka Imperial Borderlands. *Latin American Antiquity* 15:389–418.
2008 Dis-Embedded Centers and Architecture of Power in the Fringes of the Inka Empire: New Perspectives on Territorial and Hegemonic Strategies of Domination. *Journal of Anthropological Archaeology* 27(1):63–81.
2016 *Southeast Inka Frontiers: Boundaries and Interactions*. University Press of Florida, Gainesville.

Anderson, Amber
2014 *More than Forts: A Study of High Elevation Enclosures within the Pambamarca Fortress Complex, Ecuador*. PhD dissertation, Department of Anthropology, University at Buffalo, State University of New York, Buffalo.

Arkush, Elizabeth
2011 *Hillforts of the Ancient Andes: Colla Warfare, Society, and Landscape*. University Press of Florida, Gainesville.

Athens, J. Stephen
1978 Evolutionary Process in Complex Societies and the Late Period-Cara Occupation of Northern Highland Ecuador. PhD dissertation, Department of Anthropology, University of New Mexico, Albuquerque.
1992 Ethnicity and Adaptation: The Late Period-Cara Occupation in Northern Highland Ecuador. In *Resources, Power, and Interregional Interaction*, edited by Edward M. Schortman and Patricia A. Urban, pp. 193–219. Plenum Press, New York.

Downloaded from https://www.cambridge.org/core. University of New England, on 28 Mar 2019 at 21:59:08, subject to the Cambridge Core terms of use, available at https://www.cambridge.org/core/terms. https://doi.org/10.1017/laq.2018.80

2003 Inventory of Earthen Mound Sites, Northern Highland Ecuador. Manuscript on file, National Institute of Cultural Patrimony. Quito, Ecuador.

Betanzos, Juan de
1987[1551–1557] *Suma y Narración de los Incas*. Transcripción, notas y prólogo de María del Carmen Martín Rubio. En conmemoración del V Centenario del Descubrimiento de América, Ediciones Atlas, Madrid.
1996[1557] *Narratives of the Incas*. Translated and edited by Roland Hamilton and Dana Buchanan. University of Texas Press. Austin.

Bram, Joseph
1941 *An Analysis of Inca Militarism*. J. J. Augustin, New York.

Bray, Tamara L.
1991 The Effects of Inca Imperialism on the Northern Frontier. PhD dissertation, Department of Anthropology, Binghamton University, State University of New York, Binghamton.
1992 Archaeological Survey in Northern Highland Ecuador: Inca Imperialism and the País Caranqui. *World Archaeology* 24:218–233.
2008 Chiefdoms of the Ecuadorian Highlands. In *Handbook of South American Archaeology*, edited by Helaine Silverman and William Isbell, pp. 527–543. Springer, New York.
2015 At the End of Empire: Imperial Advances on the Northern Frontier. In *The Inka Empire: A Multidisciplinary Approach*, edited by Izumi Shimada, pp. 325–344. University of Texas Press, Austin.

Bray, Tamara L., and José Echeverría
2014 The Late Imperial Site of Inca-Caranqui, Northern Highland Ecuador: At the End of Empire. *Ñawpa Pacha, Journal of Andean Archaeology* 34:177–199.

Cabello Valboa, Miguel
1951[1586] *Miscelánea antártica: una historia del Perú antiguo*. Instituto de Etnologia, Facultad de Letras, Universidad Nacional Mayor de San Marcos, Lima.

Caillavet, Chantal
2000 Los cacicazgos prehispánicos del norte del Ecuador: asentamientos y distribución demográfica. In *Etnias del Norte: etnohistoria e historia de Ecuador*, edited by Chantal Caillavet, pp. 139–158. Editorial Abya Yala, Quito.

Cieza de León, Pedro de
1959[1553] *The Incas of Pedro Cieza de León*. Translated by Harriet de Onis and edited by Victor Wolfgang von Hagen. University of Oklahoma Press, Norman.

Cobo, Bernabé
1979[1653] *History of the Inca Empire: An Account of the Indians' Customs and Their Origin, Together with a Treatise on Inca Legends, History, and Social Institutions*. University of Texas Press, Austin.

Cordero Ramos, Maria Auxiliadora
1998 The Development of Social Complexity in the Northern Highlands of Ecuador: Cayambe, Pichincha Province. PhD dissertation, Department of Anthropology, University of Pittsburgh, Pennsylvania.

Currie, Elizabeth J.
2001 A Late Period Caranqui Chiefdom in the Northern Highlands of Ecuador: Archaeological Investigations at Hacienda Zuleta. *Internet Archaeology* 10. https://doi.org/10.11141/ia.10.5, accessed September 10, 2017.

D'Altroy, Terence N.
1992 *Provincial Power in the Inka Empire*. Smithsonian Institution Press, Washington, DC.
2002 *The Incas*. Blackwell Publishing, Malden, Massachusetts.

D'Altroy, Terence, and Katharina Schreiber
2004 Andean Empires. In *Andean Archaeology*, edited by Helaine Silverman, pp. 255–279. Blackwell Publishing, Malden, Massachusetts.

de la Vega, Garcilaso
1961 *The Incas: Royal Commentaries of Garcilaso the Inca*. Edited by Alain Gheerbrant. Orion Press, New York.

DeMarrais, Elizabeth, Luis Jaime Castillo, and Timothy Earle
1996 Ideology, Materialization, and Power Strategies. *Current Anthropology* 37:15–31.

Espinosa Soriano, Waldemar
1988 *Los Cayambes y Caranques: Siglos XV–XVI: El testimonio de la ethnohistoría*. 3 vols. Colección Curiñán. Instituto Otavaleño de Antropología, Ecuador.

Fox, Robin
2011 *The Tribal Imagination: Civilization and the Savage Mind*. Harvard University Press, Cambridge, Massachusetts.

Fresco, Antonio
2004 *Ingañán: La red vial del imperio inca en los Andes ecuatoriales*. Colección de Antropología Ecuatoriana. Ediciones Banco Central de Ecuador, Quito.

Fresco, Antonio, Manuel Colima, and Gustavo Espíndola
1990 *Proyecto Arqueológico "El Quito de los Incas" Octava Parte: Informe excavaciones Pucará Quitoloma (1990)*. Banco Central del Ecuador, Quito.

Fries, Eric C.
2010 Maize at Molino Loma: Imperial Inka Infrastructure on the Northern Ecuadorian Frontier. Master's thesis, Department of Anthropology, University of California, Los Angeles.

Guaman Poma de Ayala, Felipe
1980 *La Nueva Corónica y Buen Gobierno*. Translated by Jorge L. Urioste. Siglo Veintiuno, Mexico City.

Hassig, Ross
1988 *Aztec Warfare: Imperial Expansion and Political Control*. University of Oklahoma Press, Norman.

Hyslop, John
1990 *Inka Settlement Planning*. University of Texas Press, Austin.

Jijón y Caamaño, Jacinto
1914 *Contribución al Conocimiento de los Aborigenes de la Provincia de Imbabura*. Blas y Cia, Madrid.
1952 *Anthropología Prehispánica del Ecuador*. La Prensa Católica, Quito.

Julien, Catherine
1993 Finding a Fit: Archaeology and Ethnohistory of the Incas. In *Provincial Inca: Archaeological and Ethnohistorical Assessment of the Impact of the Inca State*, edited by Michael Malpass, pp. 177–233. University of Iowa Press, Iowa City.
2000 *Reading Inca History*. University of Iowa Press, Iowa City.

Keeley, Lawrence, Marisa Fontana, and Russell Quick
2007 Baffles and Bastions: The Universal Features of Fortifications. *Journal of Archaeological Research* 15 (1):55–95.

Khatchadourian, Lori
2016 *Imperial Matter: Ancient Persia and the Archaeology of Empires*. University of California Press, Oakland.

Downloaded from https://www.cambridge.org/core. University of New England, on 28 Mar 2019 at 21:59:08, subject to the Cambridge Core terms of use, available at https://www.cambridge.org/core/terms. https://doi.org/10.1017/laq.2018.80

Lippi, Ronald
1998 *Una Exploración Arqueológica del Pichincha Occidental, Ecuador*. Museo Jacinto Jijón y Caamaño, Pontifica Universidad Católica del Ecuador, Quito.
Luttwak, Edward
1976 *The Grand Strategy of the Roman Empire: From the First Century AD to the Third*. John Hopkins University Press, Baltimore.
Malpass, Michael, and Sonia Alconini (editors)
2010 *Distant Provinces in the Inka Empire: Toward a Deeper Understanding of Inka Imperialism*. University of Iowa Press, Iowa City.
Meddens, Frank M
1997 Function and Meaning of the Usnu in Late Horizon Peru. *Tawantinsuyu: una revista internacional de estudios inkas* 3:4–14.
Meddens, Frank M., Colin McEwan, and Cirilo Vivanco Pomacanchari
2010 Inca "Stone Ancestors" in Context at a High-Altitude Usnu Platform. *Latin American Antiquity* 21:173–194.
Meyers, Albert
1981 Análisis de la Cerámica de Cochasquí. In *Cochasquí: Estudios Arqueológicos*, edited by Udo Oberem, pp. 219–285. Colección Pendoneros, Instituto Otavaleño de Antropología, Otavalo, Ecuador.
2007 Toward a Reconceptualization of the Late Horizon and the Inka Period: Perspectives from Cochasquí, Ecuador, and Samaipata, Bolivia. In *Variations in the Expression of Inka Power: A Symposium at Dumbarton Oaks, 18 and 19 October 1997*, edited by Richard L. Burger, Craig Morris, and Ramiro Matos Mendieta, pp. 223–254. Harvard University Press, Cambridge, Massachusetts.
Montesinos, Fernando de
1957[1644] Memorias antiguas historiales y políticas del Peru (17th century). *Revista del Museo e Instituto Arqueológico, Universidad Nacional del Cuzco* 16–17:1–114.
2010 *The Quito Manuscript: An Inca History Preserved by Fernando de Montesinos*. Yale Publications in Anthropology, Number 88. Yale University Press, New Haven.
Moore, Jerry D.
1996 The Archaeology of Plazas and the Proxemics of Ritual: Three Andean Traditions. *American Anthropologist* 98:789–802.
Morris, Craig
1982 The Infrastructure of Inka Control in the Peruvian Central Highlands. In *The Inca and Aztec States 1400–1800*, edited by George Collier, Renato Rosaldo, and John Wirth, pp. 153–171. Academic Press, New York.
Murra, John V.
1947 The Historic Tribes of Ecuador. In *Handbook of South American Indians*, edited by Julian Steward, pp 785–822. Smithsonian Institution, Bureau of American Ethnology, Washington, DC.
Oberem, Udo, Wolfgang Wurster, Roswith Hartmann, and Jurgen Wentscher
1969 La fortaleza de montaña de Quitoloma en la sierra septentrional del Ecuador. *Boletín de la Academia Nacional de Historia* 114:196–205.
Ogburn, Dennis, Samuel Connell, and Chad Gifford
2009 Provisioning of the Inka Army in Wartime: Obsidian Procurement in Pambamarca, Ecuador. *Journal of Archaeological Science* 36:740–751.
Plaza Schuller, Fernando
1976 *La Incursión Inca en el Septentrión Andino Ecuatoriano: Antecedentes arqueológicos de la convulsiva situación de contacto cultural. Primer informe preliminar*. Instituto Otavaleño de Antropología, Otavalo, Ecuador.
1977 *El complejo de fortalezas de Pambamarca: contribución al estudio de la arquitectura militar prehispánica en la sierra norte del Ecuador*. Instituto Otavaleño de Antropología, Otavalo, Ecuador.
1978 El complejo de fortalezas de Pambamarca. *Boletín Histórico de las Fuerzas Armadas* 5/6:81–181.
Ramón, Gabriel
2017 Shaping Precolonial Concepts in the Andes: The *Ushnu* for Llocllayhuancupa (Huarochirí, Lima). *Latin American Antiquity* 28:288–307.
Ramón Valarezo, Galo
1987 *La Resistencia Andina: Cayambe, 1500–1800*. Centro Andino de Acción Popular, Quito.
Rowe, John H.
1945 Absolute Chronology in the Andean Area. *American Antiquity* 3:265–284.
1947 Inca Culture at the Time of the Spanish Conquest. In *Handbook of South American Indians*, Vol. 2, edited by Julian H. Steward, pp. 183–330. Smithsonian Institution, Bureau of American Ethnology, Washington, DC.
Salomon, Frank
1986 *Native Lords of Quito in the Age of the Incas: The Political Economy of North Andean Chiefdoms*. Cambridge University Press, New York.
Sarmiento de Gamboa, Pedro
1960[1572] *Historia Indica*. Biblioteca de Autores Españoles. Ediciones Atlas, Madrid.
2007[1572] *The History of the Incas*. Translated and edited by Brian S. Bauer and Vania Smith. University of Texas Press, Austin.
Shimada, Izumi (editor)
2015 *The Inka Empire: A Multidisciplinary Approach*. University of Texas Press, Austin.
Sistrunk, Hannah
2010 Road to Empire: Documenting an Inca Road in Northern Ecuador. *Ñawpa Pacha, Journal of Andean Archaeology* 30:189–208.
Squier, Ephraim George
1877 *Peru: Incidents of Travel and Exploration in the Land of the Incas*. Harper Brothers, New York.
Staller, John E.
2008 Dimensions of Place: The Significance of Centers to the Development of Andean Civilization: An Exploration of the *Ushnu* Concept. In *Pre-Columbian Landscapes of Creation and Origin*, edited by John E. Staller, pp. 269–313. Springer, New York.
Stanish, Charles
2001 Regional Research on the Inca. *Journal of Archaeological Research* 9:213–241.
Stoler, Ann Laura
2008 Imperial Debris: Reflections on Ruins and Ruination. *Cultural Anthropology* 23:191–219.
Sullivan, Mary C.
2007 Before the Inka to after the Spanish: Interpreting Different Cultural Patterns Using GPR in Pambamarca, Ecuador. Master's thesis, Department of Anthropology, University of Mississippi, Oxford.
Topic, John
1989 The Ostra Site: The Earliest Fortified Site in the New

Downloaded from https://www.cambridge.org/core. University of New England, on 28 Mar 2019 at 21:59:08, subject to the Cambridge Core terms of use, available at https://www.cambridge.org/core/terms. https://doi.org/10.1017/laq.2018.80

World? In *Cultures in Conflict: Current Archaeological Perspectives*, edited by Diana Claire Tkaczuk and Brian C. Vivian, pp. 215–228. University of Calgary Archaeological Association, Canada.

Uhle, Max
1926 Bibliografía sobre etnología y arqueología del Ecuador. *Boletín de la Biblioteca Nacional de Quito, Nueva Serie* 7:435–446.
1930 El desarrollo de la prehistoria ecuatoriana en los primeros cien años de la república. In *El Ecuador en cien años de independencia 1830-1930*, Tomo 1, edited by J. Gonzalo Orellana, pp. 1–22. Escuela Tipográfica Salesiana, Quito.
1939 Las Ruinas de Cochasquí. *Boletín de la Academia Nacional de Historia* 18:5–14.
1960[1925] *Estado actual de la prehistoria ecuatoriana*. Lecturas populares no. 7. Casa de la Cultura Ecuatoriana, Quito.

Williams, Raymond
1978 *Marxism and Literature*. Oxford University Press, New York.

Submitted December 9, 2017; Revised May 31, 2018; Accepted December 1, 2018

Downloaded from https://www.cambridge.org/core. University of New England, on 28 Mar 2019 at 21:59:08, subject to the Cambridge Core terms of use, available at https://www.cambridge.org/core/terms. https://doi.org/10.1017/laq.2018.80
